# A pre-post intervention study to improve fall risk assessment in older hospitalised adults: the STROLL study

**DOI:** 10.1186/s12877-025-06817-5

**Published:** 2025-11-29

**Authors:** Lucy Bolt, Anita Steck, Pascal Leist, Noël Hauri, Livia Grimm, Marie Méan, Carole E. Aubert

**Affiliations:** 1 Department of General Internal Medicine, Inselspital, Bern University Hospital, University of Bern, Bern, Switzerland; 2 Department of Medicine, Division of Internal Medicine, Lausanne University Hospital, CHUV, University of Lausanne, Lausanne, Switzerland; 3 Insel Data Science Center (IDSC), Inselspital, Bern University Hospital, Bern, Switzerland; 4 Institute of Primary Health Care (BIHAM), University of Bern, Bern, Switzerland

**Keywords:** Fall risk assessment, Older patients, Hospital, Pre-post intervention, Fall prevention

## Abstract

**Background:**

Approximately 25–30% of individuals aged ≥ 65 years fall yearly. A history of falls is a major and easy to assess risk factor predicting future falls. As systematic assessment is often lacking, we developed an interprofessional quality improvement (QI) intervention, based on this major risk factor, to improve documentation of falls history in older adults hospitalised on general internal medicine wards and tested its impact in a pre-post intervention study.

**Methods:**

This pre-post intervention study was conducted on all general internal medicine wards of two Swiss university (tertiary) hospitals from 08/2022 to 11/2023 and included a four-month observational pre-intervention, a nine-month intervention, and a three-month observational post-intervention period. The intervention was provided to residents and nurses and included an e-learning session, an oral presentation, and for nurses, monthly reminders using quizzes. Its effect was assessed among all patients aged ≥ 65 years. We used logistic regression to assess the association between the intervention period and documented falls history and, among those who had a documented falls history, compared the occurrence of patient-related outcomes (prescription of in-hospital physiotherapy, discharge to rehabilitation, emergency room visit or readmission after three months) between patients who did vs. who did not have a fall in the last 12 months.

**Results:**

The intervention’s effect was assessed among 6864 patients (mean age 79.2 [± 7.9] years, 45.7% female). The odds of a documented falls history was lower during the pre-intervention than during the intervention period (OR 0.56, 95% confidence interval [CI] 0.50–0.63), while there was no significant difference between the post-intervention and the intervention period (OR 1.11, 95% CI 0.97–1.26). Among patients who had a documented falls history, the prescription of physiotherapy (83% vs. 70%, *p* < 0.001) and discharge to a rehabilitation centre (12% vs. 9%, *p* < 0.02) were higher in patients who suffered from a fall within the last 12 months vs. those who did not.

**Conclusion:**

The QI intervention was successful in increasing the documentation of falls history. This intervention, which can easily be implemented in everyday clinical practice, is an important step to help improve care of patients at higher risk of falling.

**Supplementary Information:**

The online version contains supplementary material available at 10.1186/s12877-025-06817-5.

## Background

Falls are common in older adults, and the risk of falling increases with age [1]. Approximately 25–30% of adults aged 65 years or older experience a fall each year, with an increasing prevalence among those aged 70 to 80 years [2–4]. The consequences of falls are substantial, leading to reduced quality of life and functioning, as well as premature nursing home admission [5]. Moreover, injuries related to falls are the second leading cause of accidental deaths, resulting in approximately 684,000 fatalities each year [6]. Thus, falls not only have a profound impact on patients’ lives, but inflict significant social and healthcare costs, making them a critical issue to address [6, 7]. 

Multiple preventive interventions including exercises for patients and staff education, have shown to lower the risk of falls and therefore reduce fall-related injuries and consequences [8–10]. A comprehensive falls risk assessment of older hospitalised adults is pivotal to ensure a more efficient use of healthcare resources. However, unfortunately, systematic fall risk assessment is often lacking in routine clinical hospital practice [11–13]. This leaves persons who could benefit from targeted fall prevention interventions without the warranted care [14, 15]. 

Since the majority of patients in general internal medicine (GIM) are older, multimorbid, and/or frail, hospitalisation presents a valuable opportunity to assess their risk of falling. While multiple risk factors are associated with an increased risk of falling, including older age, multimorbidity, frailty, and the use of psychotropic medications (e.g., antidepressants, benzodiazepines, and antipsychotics), a positive history of falls has proven to be the strongest predictor for a future fall with a two- to three-fold increased risk [3, 5, 16–20]. Although guidelines recommend all older adults should receive advice on fall prevention and physical activity, identifying those with a positive falls history could help target personalised multidomain interventions to the most at-risk persons [21]. 

Previous quality improvement initiatives have sought to enhance fall prevention practices in hospital settings, often through staff education, workflow redesign, and multifactorial interventions [11, 22–24]. However, systematic reviews of these efforts have shown that the implementation and consistent use of standardised fall risk assessments remain suboptimal [11–13]. As a first step to enhance the systematic evaluation of fall risk, we thus developed an interprofessional quality improvement (QI) intervention for older adults hospitalised in general internal medicine wards, aiming to train nurses and residents to systematically document falls history, and provide teaching on fall-preventing interventions. We then assessed its impact in a pre-post intervention study. The intervention took place at hospital but was intended to impact on fall prevention both in hospital and in the community after hospital discharge.

## Methods

### Study design, setting and population

We conducted a pre-post intervention study on all GIM wards of two large university (tertiary) hospitals from the German- (hospital 1) and French-speaking (hospital 2) parts of Switzerland. The Department of General Internal Medicine of hospital 1 includes a total of 92 beds, with about 120 registered or licensed practical nurses and 80 residents. The Division of Internal Medicine of hospital 2 comprises a total of 170 beds, with about 320 registered or licensed practical nurses and 120 residents. Nurses have varying levels of experience in GIM, while residents typically have at least two years of work experience in GIM. The study was conducted from 08/2022 to 11/2023 and included a four-month observational pre-intervention period (01/08/2022 to 30/11/2022), a nine-month intervention period (01/12/2022 to 31/08/2023) and a three-month observational post-intervention period (01/09/2023 to 30/11/2023). The post-interventional period assessed the persistence of the intervention without actively implementing it.

The effect of the intervention was assessed among all patients aged 65 years or older who were admitted to the participating wards and who did not explicitly refuse to give consent for the reuse of their medical data for research purposes. In the participating hospitals, all patients are approached during hospitalisation by healthcare professionals and asked to provide informed written consent for re-use of their data for research purposes. They can explicitly refuse this request when signing the form. We included a three-month follow-up period after discharge, to evaluate for emergency room (ER) visits or readmission. We chose a three-month period instead of the frequently used 30 days because older patients often go to rehabilitation after acute hospitalisation and we wanted to increase the duration of time in the community covered by the follow-up period.

The study was waived from ethical approval by the local Institutional Review Boards (“Kantonale Ethikkommission Bern”) because it was considered as a quality improvement project and was thus not considered as human research according to Swiss regulation (request number 2023 − 00345). According to Swiss regulation, a specific consent is not required for data reuse. However, patients can explicitly refuse to have their data used for research, and such patients were therefore not included in the current project.

### Intervention

The STROLL QI intervention aimed to train nurses (including registered nurses and licensed practical nurses) and residents working on the participating wards to systematically take and document falls history, and provided teaching on fall-preventing interventions. The intervention was designed and coordinated by a multidisciplinary team including GIM physicians and experts in nursing. It consisted of (1) a 15-minute interactive e-learning delivered online (one version for nurses and another version for residents), (2) an in-person oral presentation delivered by a member of the multidisciplinary team, and (3) monthly reminder quizzes for nurses. The intervention did not target physiotherapists (or occupational therapists) directly, as we focused on the initial identification of patients at higher risk of fall before a referral to a physiotherapist took place.

Specifically, nurses and residents were trained on how to systematically ask three questions based on a quality indicator developed by the Swiss Society of General Internal Medicine, as an initial step in evaluating the risk of falls:[25] (1) “Did you fall in the last 12 months?”, if yes: (2) “How many times?” and (3) “How did you fall?”. In this manuscript, “documented falls history” refers to a documentation of the answer to those three questions. The content of the e-learning can be found in Additional file E-learning Nurses and Additional file E-learning Residents. We purposefully included solely these three questions, given that a history of falls is a major predictor of future falls and that we wanted to ensure an evaluation that can be implemented considering the time constraints in real-life everyday practice. Training was delivered electronically via the hospital’s learning platform, and the oral presentation was held on-site in staff meeting rooms of the participating GIM wards. Nurses and residents had to complete the e-learning at the beginning of the intervention period or when they newly joined the participating wards (new employees or after a rotation in another department). The intervention duration was six months, with ongoing reinforcement through the monthly quizzes and a retractable badge holder remembering the staff of assessing risk of falls.

After completion of the initial e-learning, a one-minute quiz was sent once a month to the nurses. The quizzes, whose content is available in Additional file Quizzes, aimed to remind nurses of the content of the e-learning. It was sent to the nurses only, because they are primarily responsible for taking falls history in the target settings. Finally, to increase adherence and fidelity to the intervention, there was a giveaway of five iPads in each hospital among all nurses who completed the e-learning and all quizzes. The intervention was not modified during the time of the project.

### Data collection

We extracted following data automatically from electronic health records: falls history documentation, age, sex, medications (using Anatomical Therapeutic Chemical [ATC] codes) and diagnoses from discharge letter (using International Classification of Disease version 10, German Modification [ICD-10-GM] codes). The documentation of falls history changed at the beginning of the intervention period in hospital 1. Fall-risk increasing diagnoses were defined as a personal history of falls, vertigo, muscle atrophy, orthostatic hypotension, ataxia, hemiplegia, polyneuropathy, syncope, gait alteration, epilepsy, arthritis, heart failure, urinary incontinence, cognitive impairment, diabetes, depression, vitamin D deficiency, Parkinson’s disease, delirium, dementia, and severe vision impairment (see Additional file 1, Text 1 for details on ICD-10-GM codes) [19, 20, 26]. Furthermore, a diagnosis of fall and fall-related injuries was extracted, based on free-text search, since the ICD-10-GM does not include a code for falls. In addition, we calculated the Charlson Comorbidity Index using the version published by Quan et al. [27] Fall-risk increasing medications were defined according to the STOPPFall criteria and included: benzodiazepines and benzodiazepine-related drugs, antipsychotics, opioids, antidepressants, anticholinergics, antiepileptics, diuretics, alpha-blockers for prostate hyperplasia, centrally-acting antihypertensives, antihistamines, vasodilators used in cardiac diseases, overactive bladder and urge incontinence medications (see Additional file 1, Text 2 for details on ATC codes) [28]. 

### Outcomes

The primary outcome was the proportion of patients aged 65 years or older admitted to the Department of General Internal Medicine of hospital 1 or to the Division of Internal Medicine of hospital 2, whose assessment of their history of falls was documented in the electronic health record.

We considered several secondary outcomes. First, we assessed the proportion of patients who experienced a fall during their initial hospitalisation. Second, we assessed the proportion of patients with an ambulatory ER visit or a hospital readmission within three months of discharge (due to feasibility issues, this was only assessed if this occurred at one of the two participating hospitals). Finally, we assessed following outcomes in patients with documented falls history: (a) the proportions of patients with a prescription of physiotherapy during hospitalisation; (b) the proportions of patients discharged to a rehabilitation facility; (c) the proportions of patients with an ER visit or readmission within three months of discharge.

### Data analysis

We presented baseline characteristics using frequencies with percentages for categorial variables, and means with standard deviations for continuous variables (normality assessed with Shapiro-Wilk test). We used logistic regression to assess the association between the intervention period and documented falls history. We conducted an analysis adjusting only for hospital, as well as an analysis additionally adjusting for variables associated with risk of fall, including sex, age, Charlson Comorbidity Index, fall-risk increasing medications at admission/during hospitalisation, and fall-risk increasing diagnoses. The reason for adjusting for those variables was that patients with other risk factors noticed by the clinicians might be more likely to receive a falls history. We analysed both hospitals together and separately, since different practices in the two hospitals that we cannot control for might have influenced our exposure and outcomes of interest. In addition, we conducted analyses stratified by sex, age (≥ 80 vs. <80 years), Charlson Comorbidity Index (≥ 6 vs. < 6), fall-risk increasing medications at admission/during hospitalisation, and fall-risk increasing diagnoses. We used the same methods to evaluate secondary outcomes.

In a subgroup analysis including only patients with documented falls history, we used chi-squared test to compare the following outcomes in patients with a “yes” with those with a “no” answer to the question about the history of falls: (1) the proportions of patients with a prescription of physiotherapy during hospitalisation; (2) the proportions of patients discharged to a rehabilitation facility; (3) the proportions of patients with a ER visit or readmission for any cause within three months of discharge.

Statistical significance was set at a two-sided alpha level of 0.05. We performed all analyses with Stata/MP 16 (StataCorp, Texas, USA).

## Results

### Study population

Among 6864 patients (3817 in hospital 1, 3047 in hospital 2), 3136 (45.7%) were female and the mean age was 79.2 (± 7.9) years. Fall risk-increasing diagnoses were identified in 5319 (77.5%) and fall risk-increasing drugs in 5602 (81.6%) patients (Table 1). Overall, 3092 patients had a documented falls history (45.0%). 59% of the staff completed the training [29].

**Table 1:** Characteristics of included patients

Characteristics of included patients	*N* = 6864
Age	79.2 (± 7.9)
Sex (female)	3136 (45.7)
Charlson Comorbidity Index	6.9 (± 3.1)
Fall risk-increasing diagnosis	5319 (77.5)
Fall risk-increasing drug at admission or during hospitalisation	5602 (81.6)
Site (hospital 1)	3817 (55.6)
Intervention period*	
pre-intervention	1647 (24.0)
intervention	3956 (57.6)
post-intervention	1261 (18.4)

Results are presented as the number of patients (%) or mean ± standard deviation

* Numbers and frequencies correspond to the total number of patients during each period

### Association between the intervention and documented falls history

**Fig. 1:**
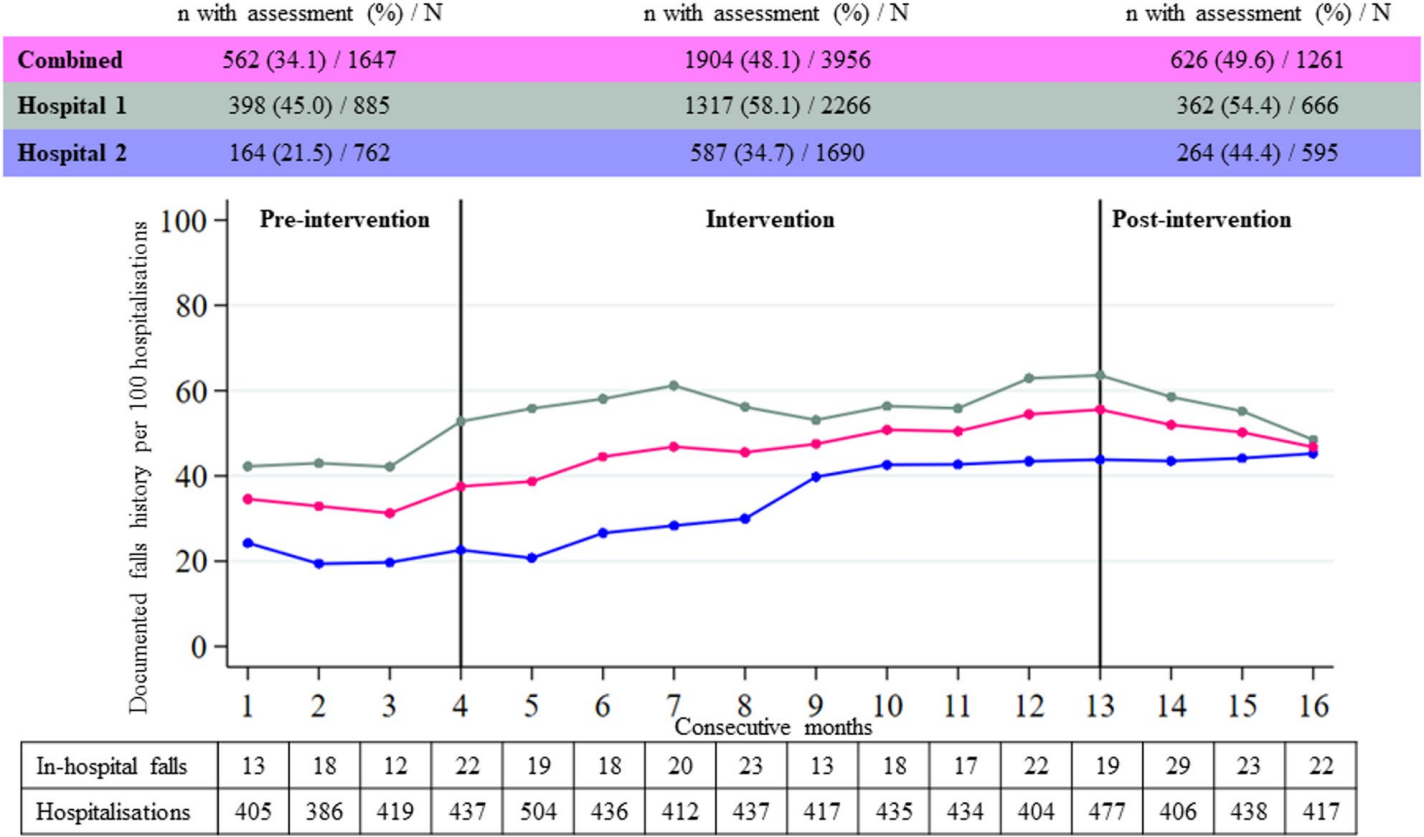
Documentation of falls history and in-hospital falls over time Legend: The figure shows the numbers of documented falls history per 100 hospitalisations for each consecutive month. The vertical black lines separate the three periods (pre-intervention, intervention, post-intervention). The upper table reports the numbers and percentages of hospitalisations with documented falls history. The numbers of patients with in-hospital falls and the number of hospitalisations for each consecutive months are reported below the graphic. Pink: both hospitals combined; teal: hospital 1; blue: hospital 2

Compared to the intervention period, we observed lower documented falls history in the pre-intervention period (both hospitals: odds ratio [OR] 0.56 [95% confidence interval [CI] 0.50–0.63]; hospital 1: OR 0.59 [95% CI 0.51–0.69]; hospital 2: OR 0.51 [95% CI 0.41–0.62]), corresponding to a 78%, 69%, and 96% increase respectively. The association sustained in the post-intervention period when both hospitals were assessed together (OR 1.11 [95% CI 0.97–1.26]) and in hospital 1 (OR 0.86 [95% CI 0.72–1.02]). The association increased in hospital 2 (OR 1.51 [95% CI 1.24–1.82]) (Fig. 1 and Table 2).

**Table 2:** Association between the intervention period and documented falls history

Exposure	Fully adjusted model^1^	Fully adjusted model, hospital 1^2^	Fully adjusted model, hospital 2^2^
	OR (95% CI)	OR (95% CI)	OR (95% CI)
Pre-intervention period^3^	0.56 (0.50–0.63)	0.59 (0.51–0.69)	0.51 (0.41–0.62)
Post-intervention period^3^	1.11 (0.97–1.26)	0.86 (0.72–1.02)	1.51 (1.24–1.82)

*Abbreviations*: *OR* Odds ratio, *CI* Confidence interval

^1^ adjusted for hospital, sex, age, Charlson Comorbidity Index, fall-risk increasing diagnosis, fall-risk increasing medication

^2^adjusted for sex, age, Charlson Comorbidity Index, fall-risk increasing diagnosis, fall-risk increasing medication

^3^compared to intervention period

Stratified analyses provided similar results (Table 3).

**Table 3:** Association between the intervention period and documented falls history, stratified analyses

Exposure	Fully adjusted model^1^	
	OR (95% CI)	
By sex	Female	Male
Pre-intervention period^2^	0.55 (0.46–0.66)	0.56 (0.48–0.66)
Post-intervention period^2^	1.13 (0.93–1.38)	1.09 (0.91–1.30)
By age	65–79	≥ 85
Pre-intervention period^2^	0.55 (0.46–0.65)	0.57 (0.48–0.68)
Post-intervention period^2^	1.16 (0.97–1.39)	1.06 (0.87–1.29)
By Charlson Comorbidity Index	< 6	≥ 6
Pre-intervention period^2^	0.56 (0.24–1.31)	0.56 (0.49–0.63)
Post-intervention period^2^	0.95 (0.30–2.96)	1.10 (0.97–1.26)
By fall risk-increasing diagnosis	Yes	No
Pre-intervention period^2^	0.58 (0.50–0.66)	0.51 (0.39–0.68)
Post-intervention period^2^	1.16 (1.00–1.35.00.35)	0.94 (0.70–1.26)
By fall risk-increasing medication	Yes	No
Pre-intervention period^2^	0.56 (0.49–0.63)	0.57 (0.43–0.76)
Post-intervention period^2^	1.14 (0.99–1.33)	0.96 (0.71–1.31)

*Abbreviations*: *OR * Odds ratio, *CI * Confidence interval

^1^ adjusted for hospital, sex, age, Charlson Comorbidity Index, fall-risk increasing diagnosis, fall-risk increasing medication

^2^ compared to intervention period

**Table 4:** Subgroup analyses – patients with documented falls history (*N* = 3092)

Outcome	Both hospitals *N* = 3092		
	Fall within last 12 months (*n* = 1452)	NO fall within last 12 months (*n* = 1640)	*p*-value^1^
	n (%)		
Prescription of physiotherapy	1200 (82.6)	1147 (69.9)	< 0.001
Discharged to rehabilitation	174 (12.0)	154 (9.4)	0.02
ER visit or readmission	555 (38.2)	617 (37.6)	0.73

*Abbreviations*: *ER * Emergency room

^1^ *p*-values were calculated using chi-square

### Subgroup analyses

Within patients who had a documented falls history, there was a statistically significant difference in the prescription of physiotherapy (82.6% vs. 69.9%, p<0.001) and discharge to a rehabilitation centre (12.0% vs. 9.4%, p=0.02) between patients who suffered from a fall within the last 12 months vs. those who did not. We did not find a difference in the occurrence of an ER visit or readmission between the two groups within three months after the initial hospitalisation (38.2% vs. 37.6%, *p*=0.73) (Table 4).

### Secondary outcomes

Compared to the intervention period, we found a 40% increase in hospitalisations with documented in-hospital falls in the post-intervention period (OR 1.40 [95% CI 1.05–1.86]), but no statistically significant change in the pre-intervention period (OR 0.95 [95% CI 0.70–1.27]), corresponding to proportions of patients with documented in-hospital falls of 4.0% in the pre-intervention, 4.3% in the intervention and 5.9% in the post-intervention period. The numbers of patients with at least one in-hospital fall for each consecutive month are reported in Fig. 1. Both in the pre-intervention and in the post-intervention period, we observed less ER visits or readmissions than in the intervention period (pre-intervention: OR 0.83 [95% CI 0.73–0.94]; post-intervention period: OR 0.84 [95% CI 0.73–0.96]).

## Discussion

In this study, we found that a QI intervention compatible with everyday clinical practice, including a regular training on risk of falls and falls prevention for nurses and residents working on GIM wards, was associated with a 78% increase in falls history documentation, but still only around half of patients were asked about history of falling. Furthermore, this association persisted during the observational follow-up period. This highlights the sustained impact of the QI intervention beyond its active implementation period.

Our results are consistent with to other reports from hospital-based fall prevention programs. Previous QI studies have shown baseline rates between 20–50% and post-intervention improvements up to 60–70% [22–24]. Our post-intervention rate of approximately 50% therefore reflects a meaningful, though still improvable, uptake. Similar to other interventions, we did not find any statistically significant association with post-hospital outcomes, suggesting that our simple intervention alone may not immediately translate into improved outcomes such as a reduction in readmissions.

The success and sustainability of our intervention were likely driven by three practical and easily adoptable steps: (1) educating staff on the importance of fall risk assessments, risk factors and consequences of falls, and on preventive measures, (2) training on conducting falls history, and (3) providing regular reminders on where falls history can be documented in electronic health records. While the first step likely raised overall awareness of falls, the second and third steps were likely more crucial in improving documented falls history. They had minimal impact on workload and seamlessly integrated into routine clinical workflows. A comprehensive fall risk assessment is tedious and time-consuming [30, 31]. Simpler approaches, like we used in our study, are more likely to be integrated into daily clinical work and therefore applied to more patients [31, 32]. This streamlining can enable more targeted in-depth assessments allowing the management of patients at highest risk of falling to be more resourceful.

Interestingly, there was an increase in documented falls history during the post-intervention period in hospital 2. While documented falls history increased primarily at the beginning of the intervention period in hospital 1, in contrast, it rose mainly during the middle and towards the end of the intervention period in hospital 2. This suggests that it may have taken longer to implement the intervention, leading to a greater impact in the post-intervention period. Several factors might explain this observation. The teams in hospital 2 were larger than in hospital 1, and the person sending e-mails with the links to the e-learning sessions and reminder quizzes was hired especially for this project, while in hospital 1, it was an employee working on the wards, who was known by the recipients. Both are known factors to influence implementation of new interventions [33, 34]. Additionally, in hospital 1, the way to document falls history changed at the beginning of the intervention period, heightening additional awareness. Lastly, cultural differences may be responsible for this difference, with greater efforts to contribute in the German-speaking areas, as previously observed [35].

The overall goal of taking a falls history is to increase targeted fall prevention strategies, reduce the risk of falls and minimise associated complications [25]. Among patients with documented falls history, we observed more physiotherapy prescriptions and discharges to rehabilitation in those with a positive history of falls. We did not find a difference in ER visits or readmission. These findings suggest an appropriate response to the management of patients with a particularly increased risk of fall which includes physiotherapy and rehabilitation [7].

Our findings also align with the recommendations of the 2022 World Falls Guidelines, which state that, in hospital settings, all older adults should be viewed at high risk of falling and that a comprehensive falls risk assessment followed by multidomain interventions should be considered [21]. Although the World Falls Guidelines advise against the use of formal falls risk screening tools in this context, they emphasise the importance of systematically identifying prior falls as a key entry point to tailored care. By demonstrating that a brief, feasible intervention embedded in everyday practice can substantially increase rates of documented falls history, our study supports this early identification step and provides a feasible strategy to trigger comprehensive assessment and prevention measures in acute-care settings.

A counterintuitive finding was a lower likelihood of experiencing an in-hospital fall in the pre-intervention period, with an even higher likelihood in the post-intervention period compared to the intervention period. This observation is likely due to heightened awareness to fall-related documentation, possibly influenced by the Hawthorne effect, which has previously been reported in a pre-post intervention QI study [36]. Being aware of participating in a QI study and knowing that fall-related documentation is being observed, may have led to a more careful overall reporting of fall-related events. However, an alternative explanation could be a regression to the mean, where a lower risk for falling in the pre-intervention period was caused by random variation. It cannot be excluded that the intervention had negative impacts, for example by focusing on fall history and not addressing other risk factors that might be more amenable, such as fall-risk increasing medications, or causes of falls. However, this is rather unlikely, since the intervention provided a comprehensive training on fall risk assessment and risk factors. Given the non-randomised design, the results should nevertheless be interpreted with caution.

### Strengths and limitations

This study has several strengths. First, the intervention targeted different groups of healthcare professionals (residents and nurses) with various levels of education and experience levels. This has the potential to enhance interprofessional collaboration, which is an important factor to improve quality of care [37]. Second, the intervention was implemented in two hospitals with different department and ward sizes and different cultural backgrounds. A limitation to consider is that the study was not primarily designed to assess patient-related outcomes such as recurrent falls or fall-related adverse events, which limits the ability to draw conclusions of the effect of the intervention on these outcomes. Additionally, due to feasibility issues, we only considered ER visits and readmissions within three months to the participating hospitals and we were not able to distinguish any ER visits and readmission from fall-related ER visits and readmissions. Although we therefore might have missed events, we expect this to be consistent across all periods, so that the comparison of the proportions of ER visits and readmissions will still be reliable. And lastly, since our study was not a randomised controlled trial, it is susceptible to various biases. For instance, external factors or events beyond our intervention might have influenced documentation of falls history and the full implementation and impact of the intervention may not have been realised at the beginning of the intervention period [38].

## Conclusion

The STROLL intervention, which can be easily implemented in everyday clinical practice, was associated with an improvement in documentation of falls history on GIM wards of two acute hospitals. This is an important step in increasing identification and optimising management of patients at particularly high risk of falling. In line with the 2022 World Falls Guidelines, our findings underscore the value of systematically documenting falls as an essential step to trigger appropriate assessment and prevention measures in hospitals.

## Supplementary Information

Additional file 1. List of ICD-10-GM codes for fall-risk increasing diagnoses, Text 2. List of ATC-codes for fall-risk increasing drugs, based on the STOPPFall criteria (doc format).

Additional file 2. E-learning for nurses (pdf format).

Additional file 3. E-learning for residents (pdf format).

Additional file 4. Reminder quizzes for nurses (pdf format).

## Data Availability

The datasets used and/or analysed during the current study are available from the corresponding author on reasonable request.

## Abbreviations

ATC: Anatomical Therapeutic Chemical
CI: Confidence interval
ER: Emergency room
GIM: General internal medicine
ICD-10-GM: International Classification of Disease version 10, German Modification
OR: Odds ratio
QI: Quality intervention
