# Supplementary material for: A pre-post intervention study to improve fall risk assessment in older hospitalised adults: the STROLL study
Source: BMC Geriatr. 2025 Nov 29;26:171. doi: 10.1186/s12877-025-06817-5 (PMC12882572; doi:10.1186/s12877-025-06817-5)
Supplement: Supplementary file 1 — Additional file 1. List of ICD-10-GM codes for fall-risk increasing diagnoses, Text 2. List of ATC-codes for fall-risk increasing drugs, based on the STOPPFall criteria (doc format). [file 12877_2025_6817_MOESM1_ESM.docx]

**ADDITIONAL FILE**

**Text 1**. List of ICD-10-GM codes for fall-risk increasing diagnoses

Vertigo: A88.1, F44.88, F45.8, H81.0, H81.1, H81.3, H81.4, H81.8, H81.9, H82, R42, N95.1, T75.2

Muscle atrophy: G12.0, G12.1, G12.2, G12.8, G12.9, G31.88, G60.0, G71.0, M62.50, M62.59, M63.89

Orthostatic hypotension: G23.8, I95.1

Ataxia: A52.1, G13.1, D64.0, G13.2, G31.88, E75.2, E79.8, F44.4, G11.0, G11.1, G11.2, G11.3, G11.8, G11.9, G60.2, R27.0

Hemiplegia: A52.1, F44.4, G40.4, G80.2, G80.8, G81.0, G81.1, G81.9, G95.88, O99.3, P11.9, P91.88, Q07.8

Polyneuropathy: G37.8, G60.0, G60.3, G60.8, G60.9, G61.1, G61.8, G62.0, G62.1, G62.2, G62.80, G62.88, G62.9, G63.0, G63.1, G63.2, G63.3, G63.4, G63.5, G63.6, G63.8, H47.2

Syncope: F48.8, G90.00, G97.1, R00.1, R05, R42, R55, R96.0, T67.1

Gait alteration: R26.0, R26.1, R26.2, R26.3, R26.8, F44.4

Epilepsy: G94.8, E10.40, E71.1, E75.4, F05.8, F06.2, F07.0, F44.5, F80.3, G11.1, G11.4, G31.81, G31.88, G40.00, G40.02, G40.08, G40.09, G40.1, G40.2, G40.3, G40.4, G40.8, G40.9

Osteoarthritis: M14.89, M15.0, M15.3, M15.4, M15.8, M15.9, M16.0, M16.1, M16.2, M16.3, M16.4, M16.5, M16.6, M16.7, M16.9, M17.0, M17.1, M17.2, M17.3, M17.4, M17.5, M17.9, M19.05, M19.07, M19.09, M19.15, M19.17, M19.19, M19.25, M19.27, M19.29, M19.85, M19.87, M19.89, M19.95, M19.97, M19.99

Heart failure: I52.0, I43.8, I01.8, I02.0, I09.8, I09.9, I11.00, I11.01, I13.00, I13.01, I13.20, I13.21, I50.00, I50.01, I50.02, I50.03, I50.04, I50.05, I50.11, I50.12, I50.13, I50.14, I50.19, I50.9, P29.0

Urinary incontinence: F98.08, N31.0, N39.3, N39.40, N39.41, N39.42, N39.43, N39.48, R32

Cognitive impairment: F06.7, F10.7, F11.7, F12.7, F13.7, F14.7, F15.7, F16.7, F17.7, F19.7, F18.7, G31.88, Q87.8, R41.3

Diabetes mellitus: E10.01, E10.11, E10.20, E10.21, E10.30, E10.31, E10.40, E10.41, E10.50, E10.51, E10.60, E10.61, E10.72, E10.73, E10.74, E10.75, E10.80, E10.81, E10.90, E10.91, E11.01, E11.11, E11.20, E11.21, E11.30, E11.31, E11.40, E11.41, E11.50, E11.51, E11.60, E11.61, E11.72, E11.73, E11.74, E11.75, E11.80, E11.81, E11.90, E11.91, E12.01, E12.11, E12.20, E12.21, E12.30, E12.31, E12.40, E12.41, E12.50, E12.51, E12.60, E12.61, E12.72, E12.73, E12.74, E12.75, E12.80, E12.81, E12.90, E12.91, E13.01, E13.11, E13.20, E13.21, E13.30, E13.31, E13.40, E13.41, E13.50, E13.51, E13.60, E13.61, E13.72, E13.73, E13.74, E13.75, E13.80, E13.81, E13.90, E13.91, E14.01, E14.11, E14.20, E14.21, E14.30, E14.31, E14.40, E14.41, E14.50, E14.51, E14.60, E14.61, E14.72, E14.73, E14.74, E14.75, E14.80, E14.81, E14.90, E14.91

Depression: F06.3, F20.4, F31.3, F31.9, F32.0, F32.1, F32.2, F32.3, F32.8, F32.9, F33.0, F33.1, F33.2, F33.3, F33.8, F33.9, F34.0, F34.1, F41.2, F42.9, F44.88, F45.2, F53.0, F92.0, O99.3

Vitamin D deficiency = E55.0, E55.9, E83.31, M83.89

Parkinson: E83.8, F02.3, G20.00, G20.01, G20.10, G20.11, G20.20, G20.21, G20.90, G20.91, G21.0, G21.1, G21.2, G21.3, G21.4, G21.8, G21.9, G22, G23.2, T42.8

Delirium: E05.5, F05.0, F05.1, F05.8, F05.9, F10.0, F10.4, F10.6, F11.4, F12.4, F13.4, F14.4, F15.4, F16.4, F17.4, F18.4, F19.4, F23.0, F23.1, F43.0, F44.88, N19, O15.9, T44.3

Dementia: F00.0, F00.1, F00.2, F00.9, F01.0, F01.1, F01.2, F01.3, F01.8, F01.9, F02.0, F02.1, F02.2, F02.3, F02.4, F02.8, F03, F05.0, F05.1, F10.7, F13.7, F18.7

Severe vision impairment: H54.1, H54.5

History of fall: R29.6, Z91.8

**Text 2**. List of ATC-codes for fall-risk increasing drugs, based on the STOPPFall criteria

Benzodiazepines and z-drugs: N05BA01, N05BA02, N05BA03, N05BA04, N05BA05, N05BA06, N05BA07, N05BA08, N05BA09, N05BA10, N05BA11, N05BA12, N05BA13, N05BA14, N05BA15, N05BA16, N05BA17, N05BA18, N05BA19, N05BA21, N05BA22, N05BA23, N05BA24, N05BA25, N05BA56, N05CD01, N05CD02, N05CD03, N05CD04, N05CD05, N05CD06, N05CD07, N05CD08, N05CD09, N05CD10, N05CD11, N05CD12, N05CD13, N05CD14, N05CD15, N05CF01, N05CF02, N05CF03, N05CF04

Antipsychotics: N05AA01, N05AA02, N05AA03, N05AA04, N05AA05, N05AA06, N05AA07, N05AB01, N05AB02, N05AB03, N05AB04, N05AB05, N05AB06, N05AB07, N05AB08, N05AB09, N05AB10, N05AC01, N05AC02, N05AC03, N05AC04, N05AD01, N05AD02, N05AD03, N05AD04, N05AD05, N05AD06, N05AD07, N05AD08, N05AD09, N05AD10, N05AE01, N05AE02, N05AE03, N05AE04, N05AE05, N05AF01, N05AF02, N05AF03, N05AF04, N05AF05, N05AG01, N05AG02, N05AG03, N05AH01, N05AH02, N05AH03, N05AH04, N05AH05, N05AH06, N05AH53, N05AL01, N05AL02, N05AL03, N05AL04, N05AL05, N05AL06, N05AL07, N05AX07, N05AX08, N05AX10, N05AX11, N05AX12, N05AX13, N05AX14, N05AX15, N05AX16, N05AX17

Antidepressants: N06AA01, N06AA02, N06AA03, N06AA04, N06AA05, N06AA06, N06AA07, N06AA08, N06AA09, N06AA10, N06AA11, N06AA12, N06AA13, N06AA14, N06AA15, N06AA16, N06AA17, N06AA18, N06AA19, N06AA21, N06AA23, N06AB02, N06AB03, N06AB04, N06AB05, N06AB06, N06AB07, N06AB08, N06AB09, N06AB10, N06AF01, N06AF02, N06AF03, N06AF04, N06AF05, N06AF06, N06AG02, N06AG03, N06AX01, N06AX02, N06AX03, N06AX04, N06AX05, N06AX06, N06AX07, N06AX08, N06AX09, N06AX10, N06AX11, N06AX12, N06AX13, N06AX14, N06AX15, N06AX16, N06AX17, N06AX18, N06AX19, N06AX21, N06AX22, N06AX23, N06AX24, N06AX25, N06AX26, N06AX27, N06AX28, N06AX29, N06AX62

Opioids: N02AA01, N02AA02, N02AA03, N02AA04, N02AA05, N02AA08, N02AA10, N02AA11, N02AA51, N02AA53, N02AA55, N02AA56, N02AA58, N02AA59, N02AA79, N02AB01, N02AB02, N02AB03, N02AB52, N02AB72, N02AC01, N02AC03, N02AC04, N02AC05, N02AC52, N02AC54, N02AC74, N02AD01, N02AD02, N02AD51, N02AE01, N02AF01, N02AF02, N02AG01, N02AG02, N02AG03, N02AG04, N02AJ01, N02AJ02, N02AJ03, N02AJ06, N02AJ07, N02AJ08, N02AJ09, N02AJ13, N02AJ14, N02AJ15, N02AJ16, N02AJ17, N02AJ18, N02AJ19, N02AJ22, N02AJ23, N02AX01, N02AX02, N02AX03, N02AX05, N02AX06, N02AX07, N02AX51

Antiepileptics: N03AA01, N03AA02, N03AA03, N03AA04, N03AA30, N03AB01, N03AB02, N03AB03, N03AB04, N03AB05, N03AB52, N03AB54, N03AC01, N03AC02, N03AC03, N03AD01, N03AD02, N03AD03, N03AD51, N03AE01, N03AF01, N03AF02, N03AF03, N03AF04, N03AG01, N03AG02, N03AG03, N03AG04, N03AG05, N03AG06, N03AX03, N03AX07, N03AX09, N03AX10, N03AX11, N03AX13, N03AX14, N03AX15, N03AX17, N03AX18, N03AX19, N03AX21, N03AX22, N03AX23, N03AX24, N03AX25, N03AX26, N03AX27, N03AX30

Diuretics: C03CA01, C03CA02, C03CA03, C03CA04, C03CB01, C03CB02, C03CC01, C03CD01, C03CX01

Alphablockers: C02CA01, C02CA02, C02CA03, C02CA04, C02CA06, G04CA01, G04CA02, G04CA03, G04CA04, G04CA51, G04CA52, G04CA53, G04CA54, G04CA55, G04CA01, G04CA02, G04CA03, G04CA04, G04CA51, G04CA52, G04CA53, G04CA54, G04CA55

Antiadrenergics: C02AA01, C02AA02, C02AA03, C02AA04, C02AA05, C02AA06, C02AA07, C02AA52, C02AA53, C02AA57, C02AB01, C02AB02, C02AC01, C02AC02, C02AC04, C02AC05, C02AC06

Antihistamines: R06AA01, R06AA02, R06AA04, R06AA06, R06AA07, R06AA08, R06AA09, R06AA10, R06AA11, R06AA52, R06AA54, R06AA56, R06AA57, R06AA59, R06AA61, R06AB01, R06AB02, R06AB03, R06AB04, R06AB05, R06AB06, R06AB07, R06AB51, R06AB52, R06AB54, R06AB56, R06AC01, R06AC02, R06AC03, R06AC04, R06AC05, R06AC06, R06AC52, R06AC53, R06AD01, R06AD02, R06AD03, R06AD04, R06AD05, R06AD06, R06AD07, R06AD08, R06AD09, R06AD52, R06AD55, R06AE01, R06AE03, R06AE04, R06AE05, R06AE06, R06AE07, R06AE09, R06AE51, R06AE53, R06AE55, R06AX01, R06AX02, R06AX03, R06AX04, R06AX05, R06AX07, R06AX08, R06AX09, R06AX11, R06AX12, R06AX13, R06AX15, R06AX16, R06AX17, R06AX18, R06AX19, R06AX21, R06AX22, R06AX23, R06AX24, R06AX25, R06AX26, R06AX27, R06AX28, R06AX29, R06AX31, R06AX32, R06AX53, R06AX58

Vasodilators: C01DA02, C01DA04, C01DA05, C01DA07, C01DA08, C01DA09, C01DA13, C01DA14, C01DA20, C01DA38, C01DA52, C01DA54, C01DA55, C01DA57, C01DA58, C01DA59, C01DA63, C01DA70, C01DB01, C01DX01, C01DX02, C01DX03, C01DX04, C01DX05, C01DX06, C01DX07, C01DX08, C01DX09, C01DX10, C01DX11, C01DX12, C01DX13, C01DX14, C01DX15, C01DX16, C01DX18, C01DX19, C01DX21, C01DX22, C01DX51, C01DX52, C01DX53, C01DX54

Anticholinergics: N04AA01, N04AA02, N04AA03, N04AA04, N04AA05, N04AA08, N04AA09, N04AA10, N04AA11, N04AA12, N04AB01, N04AB02, N04AC01, N04AC30, G04BD01, G04BD02, G04BD03, G04BD04, G04BD05, G04BD06, G04BD07, G04BD08, G04BD09, G04BD10, G04BD11, G04BD12, G04BD13, G04BD14, G04BD15
