# Supplementary material for: A pre-post intervention study to improve fall risk assessment in older hospitalised adults: the STROLL study
Source: BMC Geriatr. 2025 Nov 29;26:171. doi: 10.1186/s12877-025-06817-5 (PMC12882572; doi:10.1186/s12877-025-06817-5)
Supplement: Supplementary file 2 — Additional file 2. E-learning for nurses (pdf format). [file 12877_2025_6817_MOESM2_ESM.pdf]

The most important thing is to stand up again!

Fall prevention through three key points

Learning Goals

After completion of this e-learning, you are able to:

- Recognize and document falls that happened during the past 12 months.
- Identify the risk of future falls.
- Suggest preventive measures to hospitalized patients and their relatives.

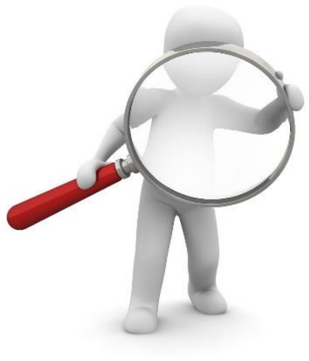

Content

- Fall risk factors in older persons
- Fall risk assessement
- Fall prevention

Recognize, document and prevent falls

Who is responsible? ALL of us!

- Patients and their relatives
- Nurses
- Nursing assistants
- Physicians
- Physiotherapists

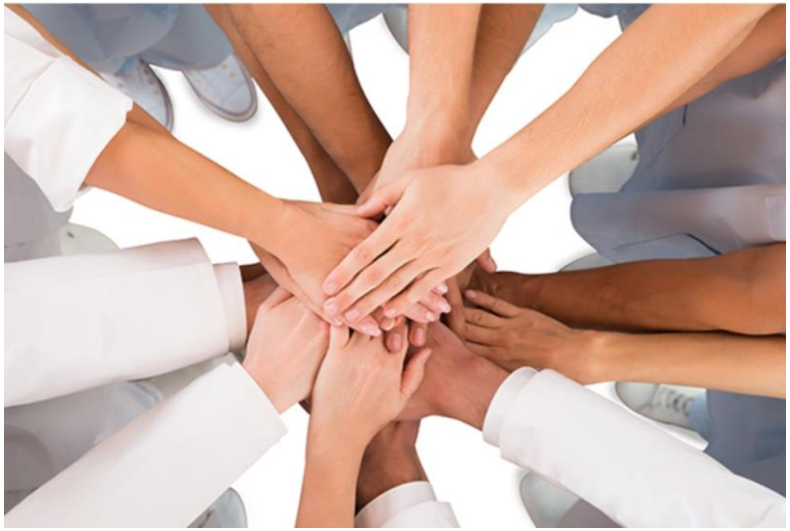

Falls, a short summary

Consequence of gait disorders

Event after which the person suddenly finds themselves on the ground

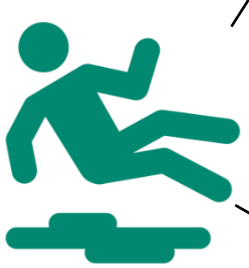

Often multifactorial and preventable

Risk increases with age

Influence on quality of life and physical health

Annual costs of two billion Swiss francs

Influence of age on the risk of falls

- The risk of falls increases exponentially after the age of 65:
    - **35%** of adults aged **≥80 years** have fallen in the past 12 months

Predisposing risk factors

- Falls within the last 12 months
- Fear of falling
- Visual impairment
- Delirium, dementia, cognitive impairment
- Depression
- Epilepsy
- Hemiplegia
- Gait and balance disorders
- Parkinson's disease
- Joint pain
- Cardiac arrhythmias
- Orthostatic hypotension
- Syncope
- Malnutrition
- Urinary incontinence

Triggering risk factors

- Inappropriate use of walking aid
- Inappropriate shoes
- Environment (e.g., insufficient light) and change of environment
- Inadequate freedom-restricting measures
- Medications that increase the risk of falls

Medications that increase the risk of falls:

1. antipsychotics
2. antidepressants
3. anticholinergics (including medication against irritable bladder)
4. antiepileptics
5. antihistamines
6. alpha blockers (as antihypertensive or against prostate hyperplasia)
7. hypnotics & sedatives
8. antihypertensives
9. vasodilators
10. diuretics
11. opioids

Question 2

The following questions are important to ask. Which of these questions should be **prioritized** upon admission in order to avoid a fall during and after hospitalization?

Only one answer is correct

1. Do you need to climb stairs to reach your apartment?

2. Did you fall in the past 12 months? If so, how often?

3. Do you need a walking aid?

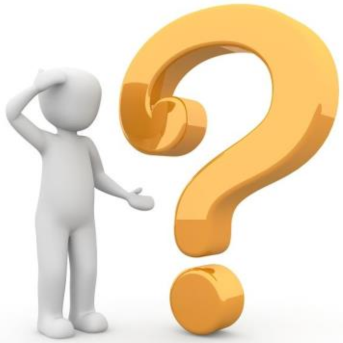

Answer to question 2

The following questions are important to ask. Which of these questions should be **prioritized** upon admission in order to avoid a fall during and after hospitalization?

Only one answer is correct

1. This answer is correct but it's not the first question which should be asked.

2. Correct!

3. This answer is correct but it's not the first question which should be asked.

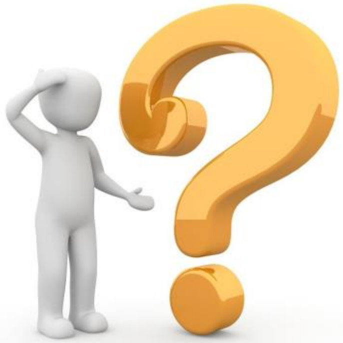

How can the risk of falls be recognized?
3 Key aspects

- Systematic questioning of individuals aged ≥65 years upon admission
- Did you **fall** in the past 12 months?

  if yes

- Details about the fall: How often and how did it happen?

And you, did you fall?

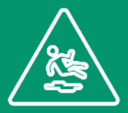

How and where to document?

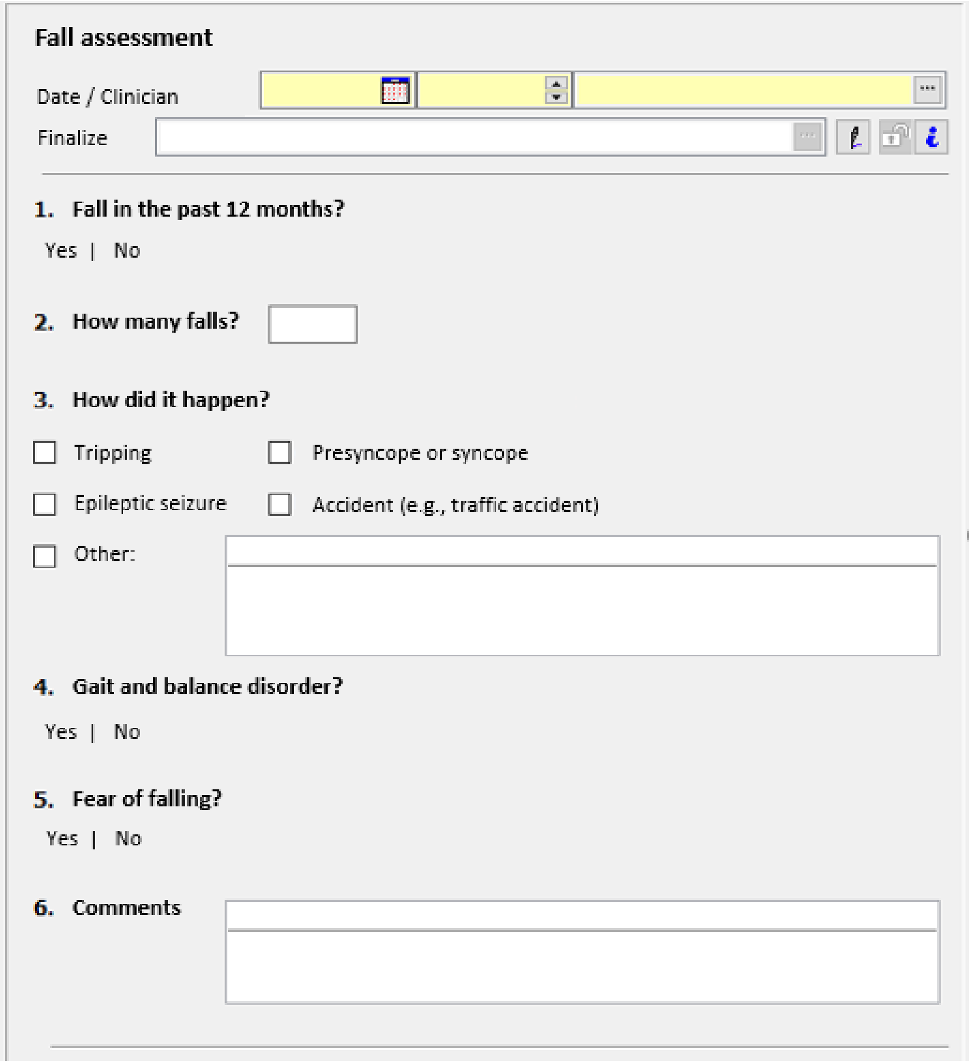

Fall assessment

Date / Clinician

Finalize

1. Fall in the past 12 months?

Yes | No

2. How many falls?

3. How did it happen?

- [ ] Tripping
- [ ] Presyncope or syncope
- [ ] Epileptic seizure
- [ ] Accident (e.g., traffic accident)
- [ ] Other:

4. Gait and balance disorder?

Yes | No

5. Fear of falling?

Yes | No

6. Comments

- Questions 1, 4 and 5 must always be answered
- Questions 2 and 3 only if “yes” to question 1

Question 2

Which of these measures can prevent a fall of older hospitalized persons?

Several answers are correct.

1. A urinary catheter that reduces movement to the bathroom.
2. Side rails to prevent a patient from standing up.
3. Not changing pre-existing medication.
4. Encouraging exercise during the rounds, independently of physiotherapy.
5. Not prescribing benzodiazepines.

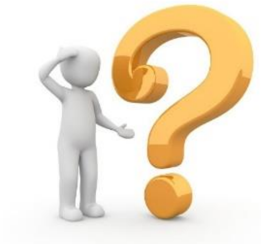

Answer to question 2

Which of these measures can prevent a fall of older hospitalized persons?

Several answers are correct.

1. This is not a measure of fall prevention.

2. This is not a measure of fall prevention.

3. This is not a measure of fall prevention, the current medication should always be reviewed.

4. Correct!

5. Correct!

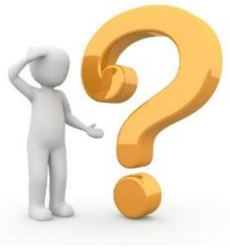

Measures to prevent falls

The measures to prevent falls must be tailored individually. Here are some examples:

- Encourage movement throughout the day without waiting for physiotherapy.
- Review medication: Active ingredient, indication, dosage, time of administration.
- Adapt food to nutritional needs.
- Evaluate hazards in the environment (light, objects on the ground).
- Provide an alarm system (e.g., patient bell).

Measures to prevent falls

- Podiatry and adapted shoes: Closed, with adjustable fasteners, correct size, soft material.
- Eyeglasses, phone, assistive devices available.
- Bathroom easy to reach.
- Inquire about living conditions so that the necessary adjustments can be organized before discharge.
- Educate patients and their relatives: Ask about individual needs of patients and their relatives to prevent falls during and after hospitalization.

An important brochure

Independent until old age

Live, move, stay mobile

Swiss Council for Accident Prevention

bfu.ch

bfu bpa upi

- Available online and on the ward
- Contents:
  - Tricks
  - Self-assessment of fall risk
  - Checklist

The most important tricks

- Are you worried that you might fall? Then contact your primary care provider.
- By furnishing your home in an age-appropriate manner, you make everyday life easier and prevent falls.
- Daily exercise and a healthy diet contribute to preserving mobility and independence.

Don’t forget!

Early prevention thanks to one question that should be documented systematically:

Did you fall in the last 12 months?

↓

It affects all of us! YOU are the key!

And you, did you fall?
