# Supplementary material for: A pre-post intervention study to improve fall risk assessment in older hospitalised adults: the STROLL study
Source: BMC Geriatr. 2025 Nov 29;26:171. doi: 10.1186/s12877-025-06817-5 (PMC12882572; doi:10.1186/s12877-025-06817-5)
Supplement: Supplementary file 3 — Additional file 3. E-learning for residents (pdf format). [file 12877_2025_6817_MOESM3_ESM.pdf]

Quiz 1

Question

What are risk factors for falls?

Several answers are correct

A. Urinary incontinence
B. Fall at home two months ago
C. Fear from falling
D. Joint pain

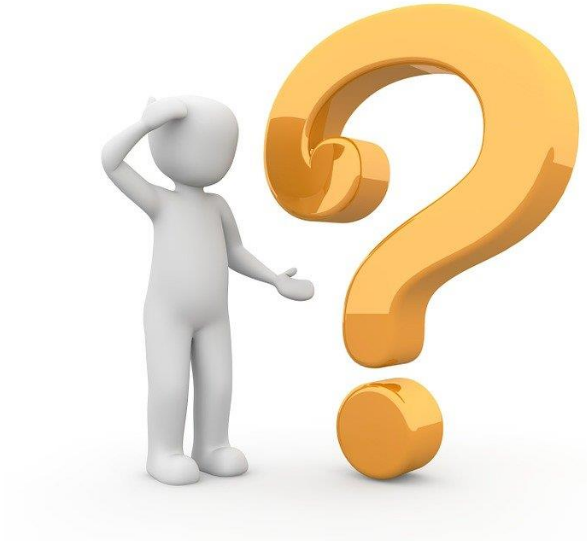

Predisposing risk factors

- Falls within the last 12 months
- Fear of falling
- Visual impairment
- Delirium, dementia, cognitive impairment
- Depression
- Epilepsy
- Hemiplegia
- Gait and balance disorders
- Parkinson's disease
- Joint pain
- Cardiac arrhythmias
- Orthostatic hypotension
- Syncope
- Malnutrition
- Urinary incontinence

Triggering risk factors

- Inappropriate use of walking aids
- Inappropriate shoes
- Environment (e.g. insufficient light) and change of environment
- Inadequate freedom-restricting measures
- Medications that increase the risk of falls

How and where to document a risk of falls

- Documentation is done by nurses in the EHR.
- Open dossier: Nursing assessement => To the right of «Habits / Needs»: click on «ST»
- Physicians also have access to the questionnaire and can make adjustments.

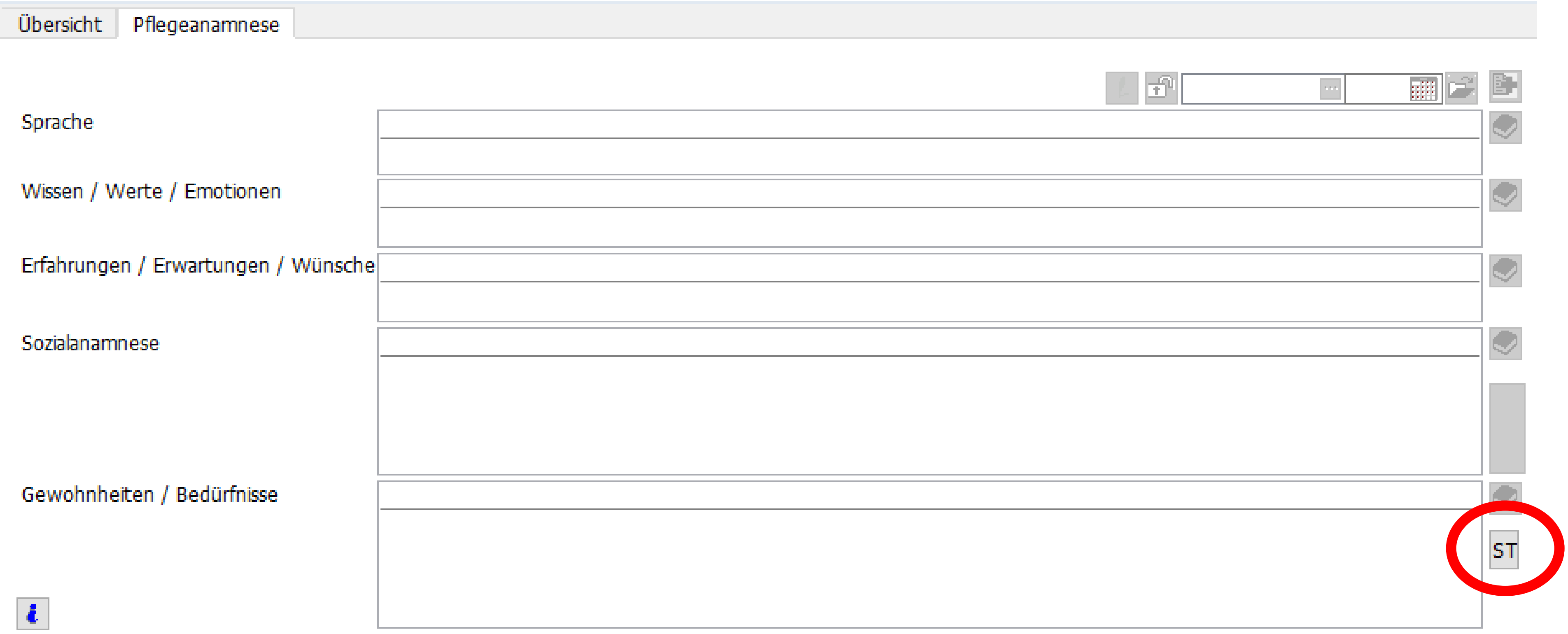

Documentation in the EHR

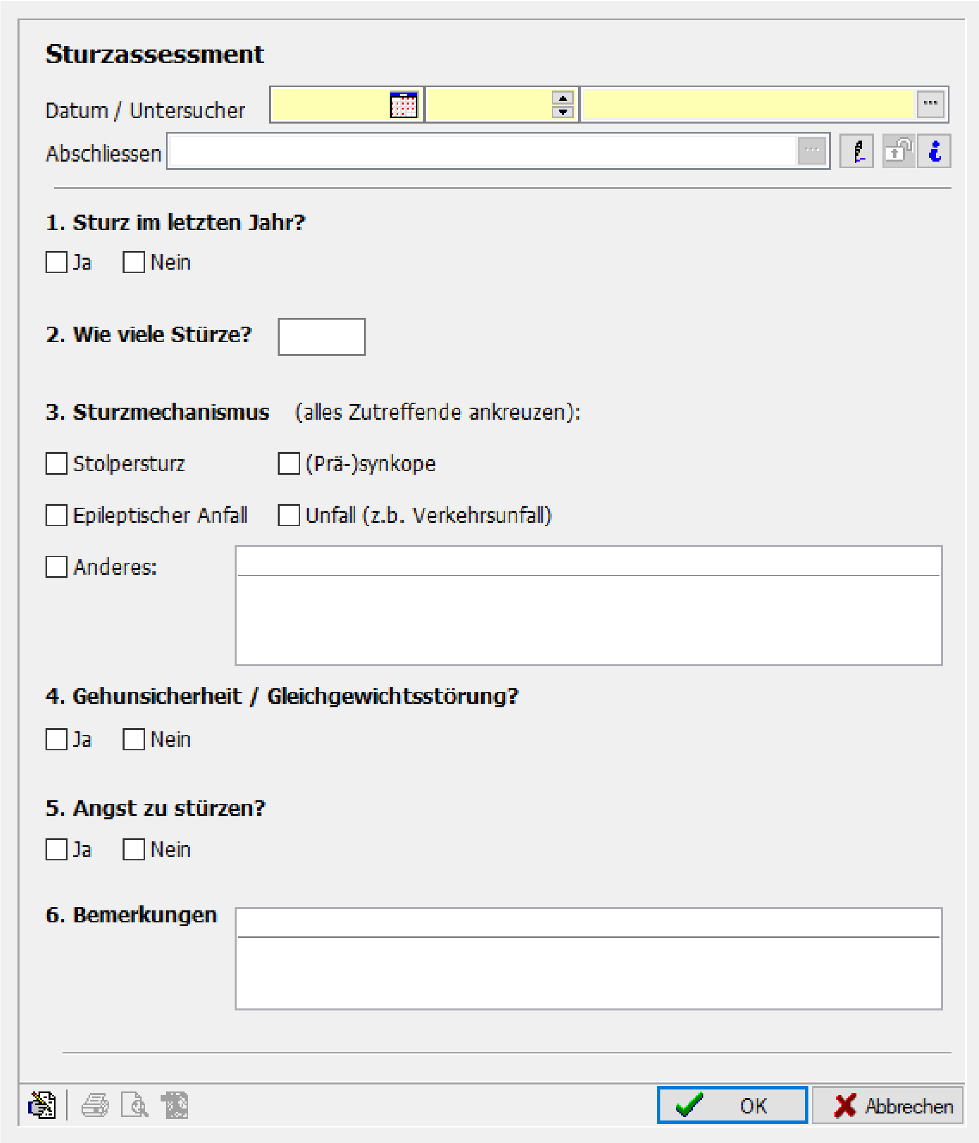

- Questions 1, 4 and 5 must always be answered
- Questions 2 and 3 only if “Yes” to question 1

Quiz 2

Question

Which drug increases the risk of falls?

Several Answers are correct

A. Lorazepam
B. Amlodipine
C. Acetaminophen
D. Ceftriaxone
E. Furosemide i.v.

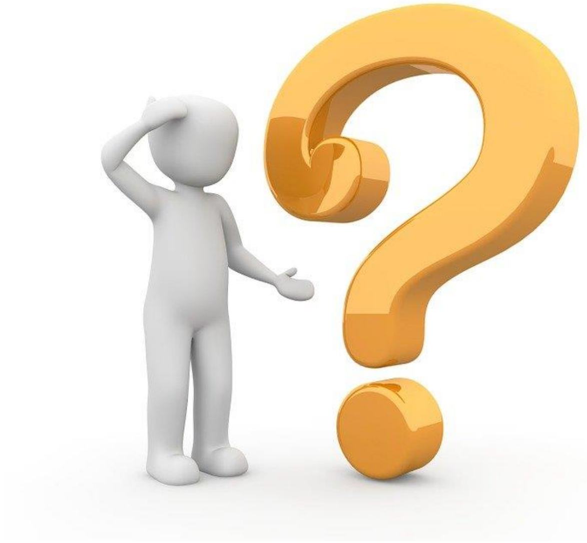

Medications that increase the risk of falls?

1. antipsychotics
2. antidepressants
3. anticholinergics (including drugs for irritable bladder)
4. antiepileptics
5. antihistamines
6. alpha blockers (antihypertensives / drugs for benign prostatic hyperplasia)
7. benzodiazepines & other sedatives
8. antihypertensives
9. vasodilators
10. diuretics
11. opioids

How and where to document a risk of falls

- Documentation is done by nurses in the EHR.
- Open dossier: Nursing assessement => To the right of «Habits / Needs»: click on «ST»
- Physicians also have access to the questionnaire and can make adjustments.

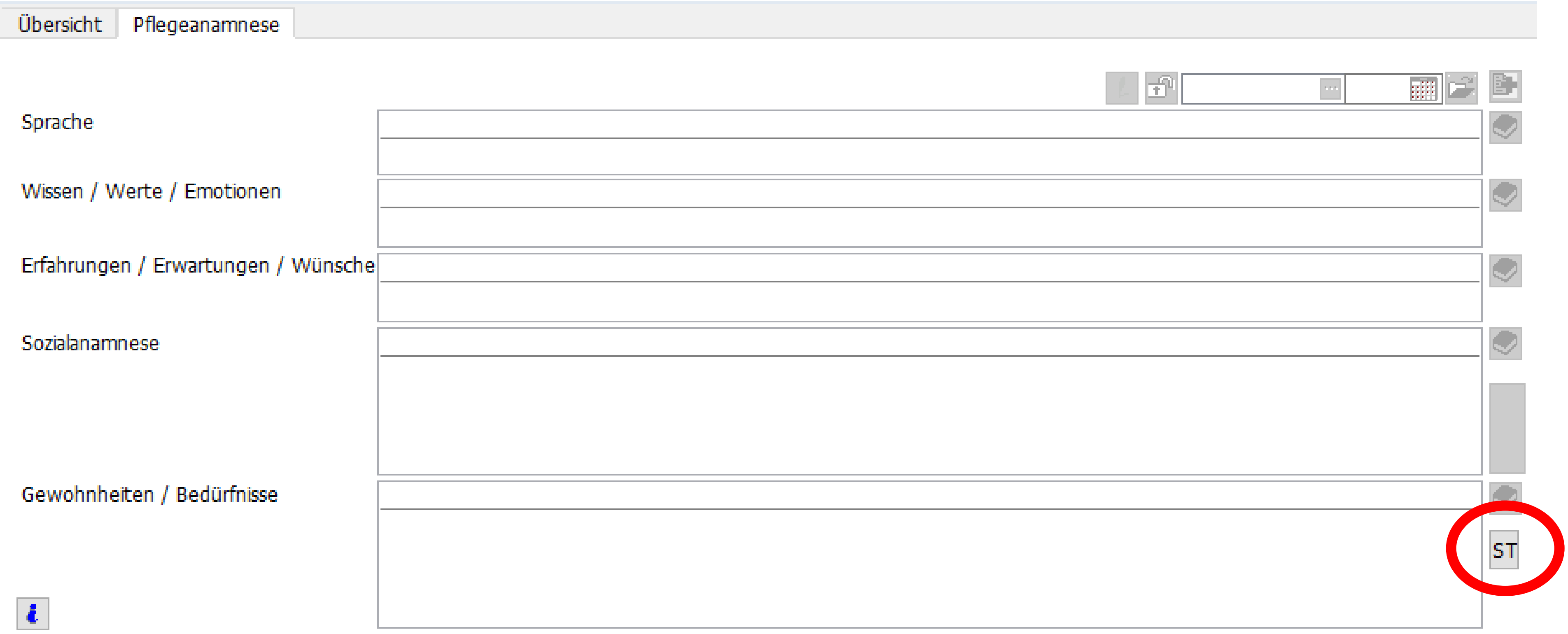

Documentation in the EHR

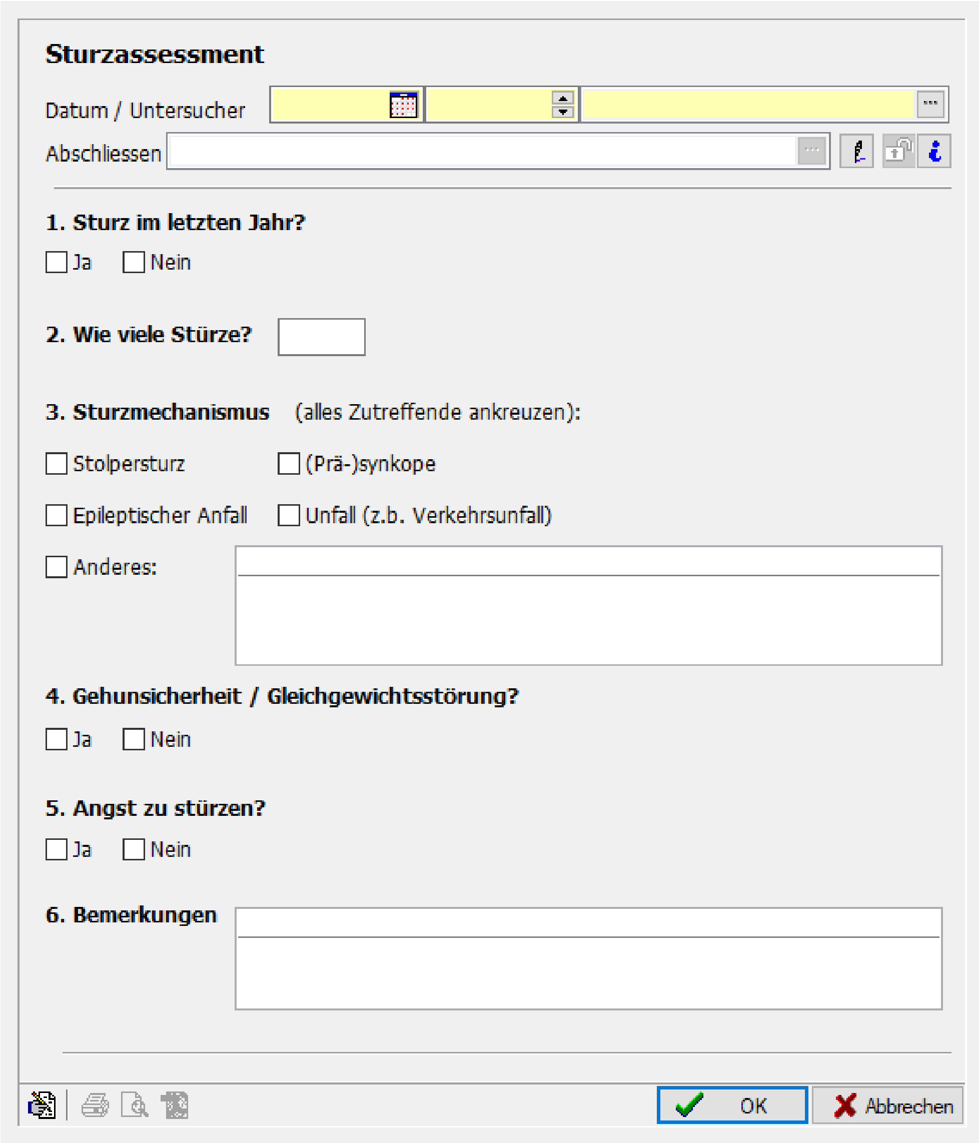

Sturzassessment

Datum / Untersucher

Abschliessen

1. Sturz im letzten Jahr?

☐ Ja ☐ Nein

2. Wie viele Stürze?

3. Sturzmechanismus (alles Zutreffende ankreuzen):

☐ Stolpersturz ☐ (Prä-)synkope

☐ Epileptischer Anfall ☐ Unfall (z.b. Verkehrsunfall)

☐ Anderes:

4. Gehunsicherheit / Gleichgewichtsstörung?

☐ Ja ☐ Nein

5. Angst zu stürzen?

☐ Ja ☐ Nein

6. Bemerkungen

OK Abbrechen

- Questions 1, 4 and 5 must always be answered
- Questions 2 and 3 only if “Yes” to question 1

Quiz 3

Question

The following questions are important to ask. Which of these questions should be **prioritized** at admission in order to avoid a fall during and after hospitalization?

Only one answer is correct

1. Do you need to climb stairs to reach your apartment?
2. Did you fall in the last 12 months?, and if so, how often?
3. Do you need a walking aid to get around?

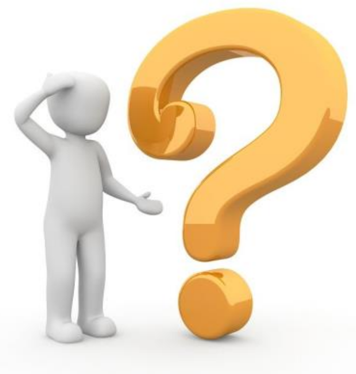

How can the risk of falls be recognized?
3 Key Aspects

- Systematic questioning of those over 65 years of age at admission
- Did you **fall** in the last 12 months?

    if yes

- Details about the fall: How often and how did it happen?

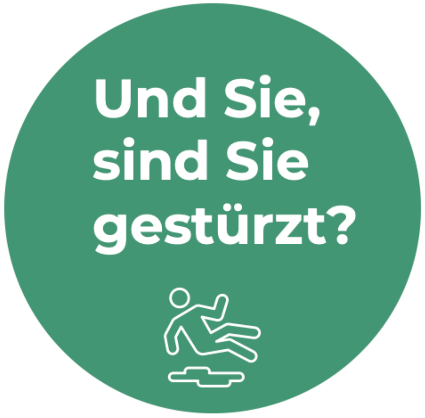

How and where to document a risk of falls

- Documentation is done by nurses in the EHR.
- Open dossier: Nursing assessement => To the right of «Habits / Needs»: click on «ST»
- Physicians also have access to the questionnaire and can make adjustments.

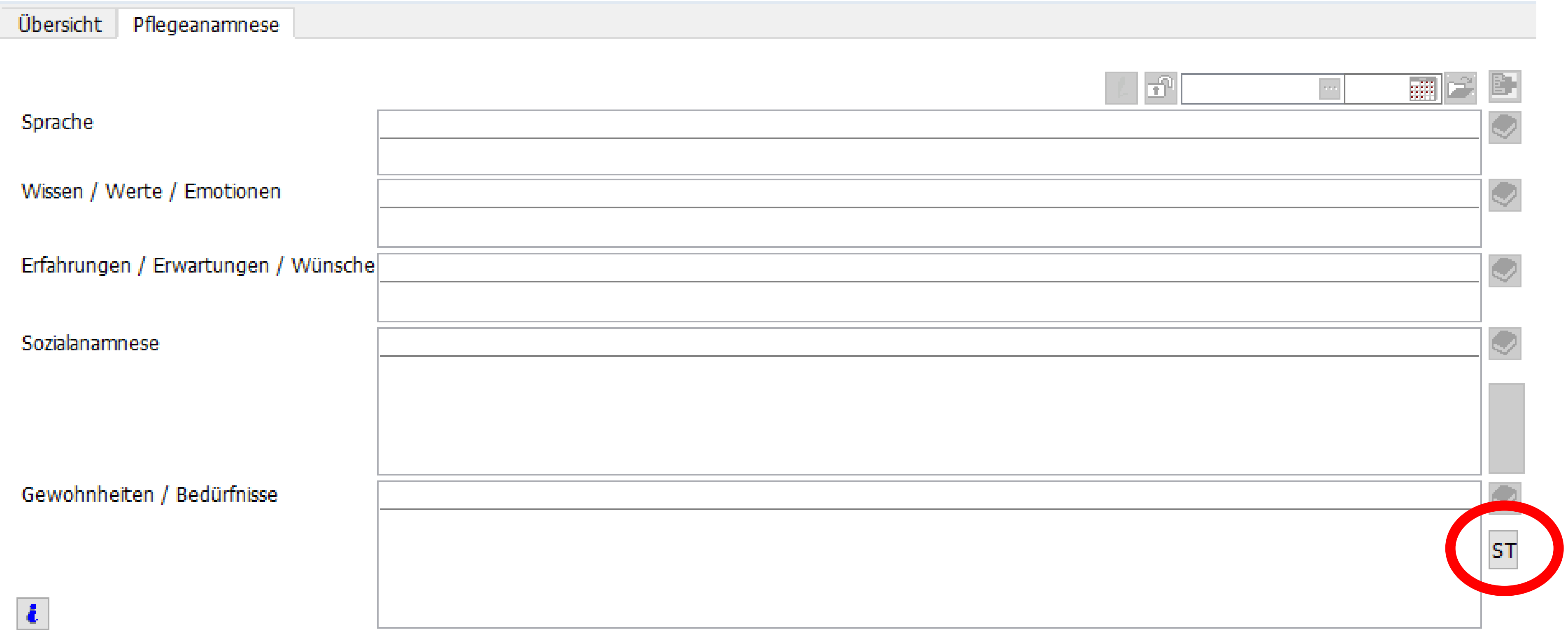

Documentation in the EHR

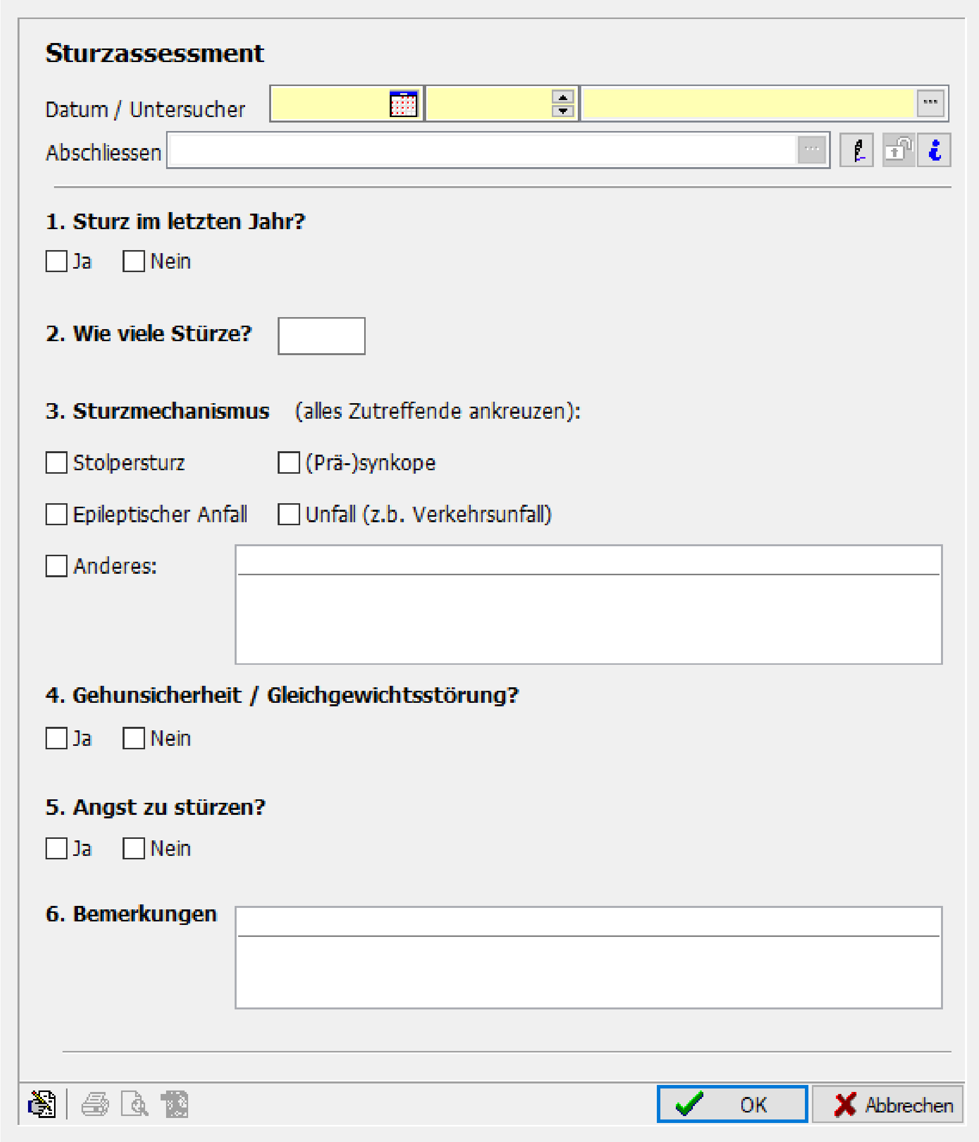

- Questions 1, 4 and 5 must always be answered
- Questions 2 and 3 only if “Yes” to question 1

Quiz 4

Question 4

Which of these measures can prevent a fall of older hospitalized persons?

One answer is correct

1) A urinary catheter that reduces movement to the toilet.
2) Encourage exercise, without waiting for physiotherapy.
3) Side rails to prevent a patient from standing up.

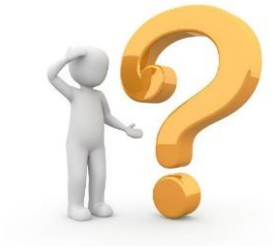

Measures to prevent falls - 1

The measures to prevent falls must be tailored individually. Here are some measures:

- Encourage movement throughout the day without waiting for physiotherapy.
- Review of medication: active ingredient, indication, dosage, time of administration.
- Adapt food to nutritional needs.
- Evaluate hazards in the environment (light, objects on the ground).
- Provide an alarm system (e.g., patient bell).

Measures to prevent falls - 2

- Podiatry and adapted shoes: Closed, with adjustable fasteners, correct size, soft material.
- Eyeglasses, telephone, assistive devices available.
- Toilet easy to reach.
- Inquire about living conditions so that the necessary adjustments can be organized before discharge home.
- Educate patients and their relatives: Ask about individual needs of patients and their relatives to prevent falls during and after hospitalization.

How and where to document a risk of falls

- Documentation is done by nurses in the EHR.
- Open dossier: Nursing assessement => To the right of «Habits / Needs»: click on «ST»
- Physicians also have access to the questionnaire and can make adjustments.

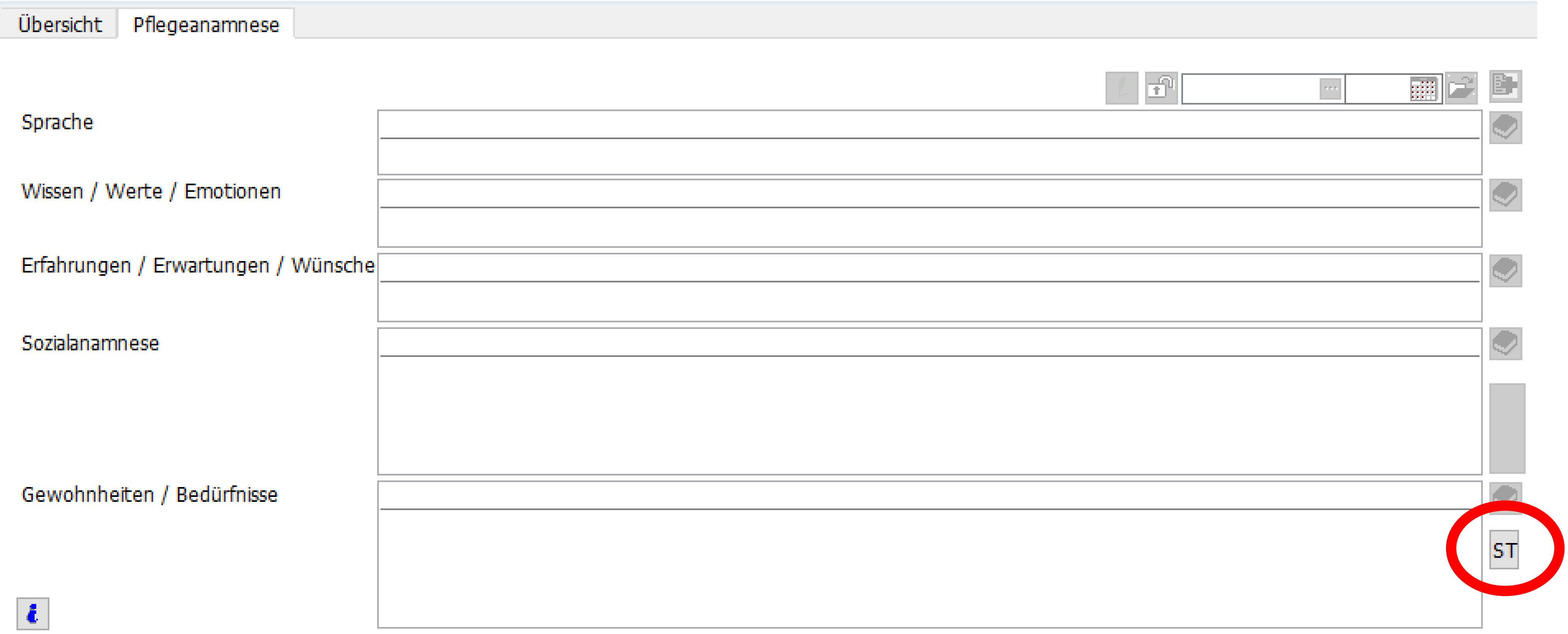

Documentation in the EHR

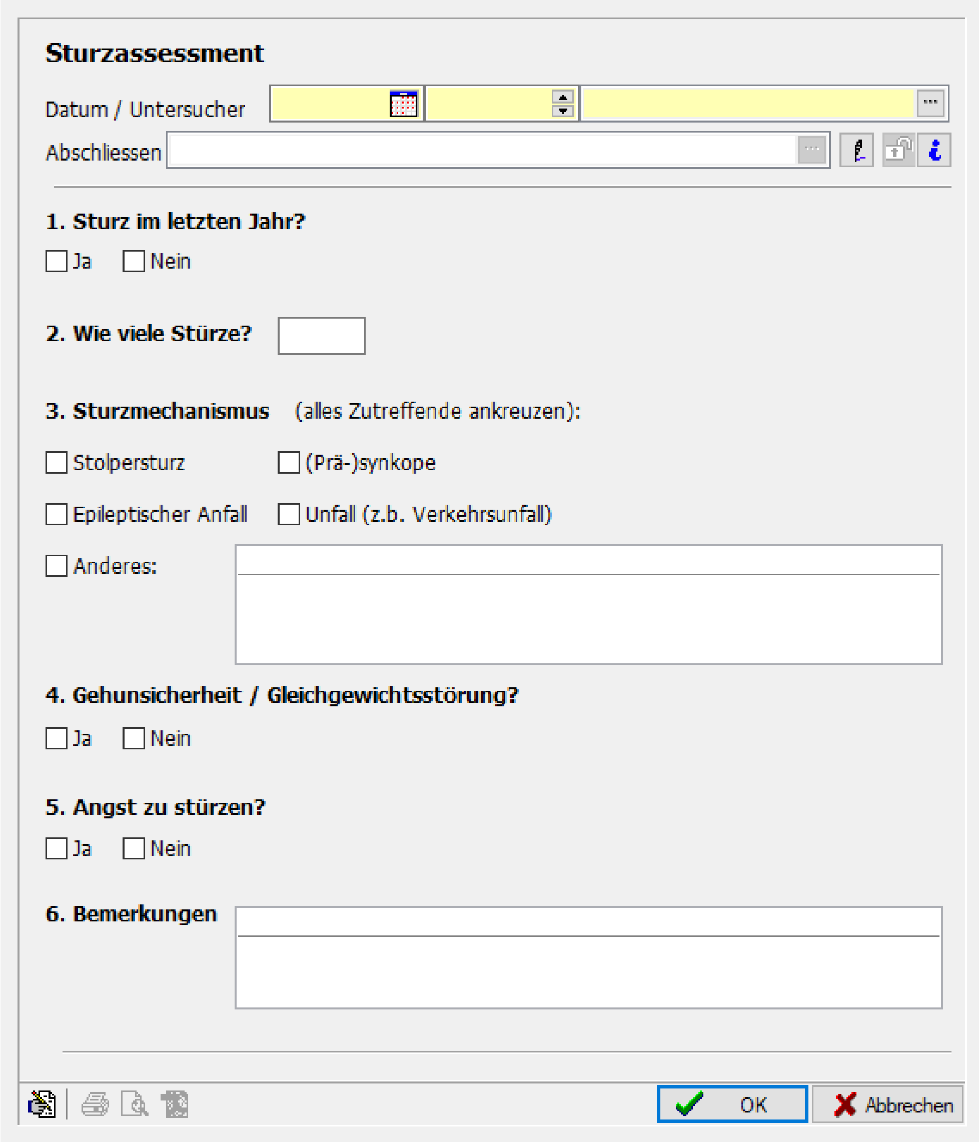

Sturzassessment

Datum / Untersucher

Abschliessen

1. Sturz im letzten Jahr?

☐ Ja ☐ Nein

2. Wie viele Stürze?

3. Sturzmechanismus (alles Zutreffende ankreuzen):

☐ Stolpersturz ☐ (Prä-)synkope

☐ Epileptischer Anfall ☐ Unfall (z.b. Verkehrsunfall)

☐ Anderes:

4. Gehunsicherheit / Gleichgewichtsstörung?

☐ Ja ☐ Nein

5. Angst zu stürzen?

☐ Ja ☐ Nein

6. Bemerkungen

OK Abbrechen

- Questions 1, 4 and 5 must always be answered
- Questions 2 and 3 only if “Yes” to question 1

Quiz 5

Question

Which footwear helps prevent falls?

Several answers are correct.

1. Open footwear that is easy to remove
2. Closed footwear
3. Footwear with non-slip soles
4. Footwear without heels

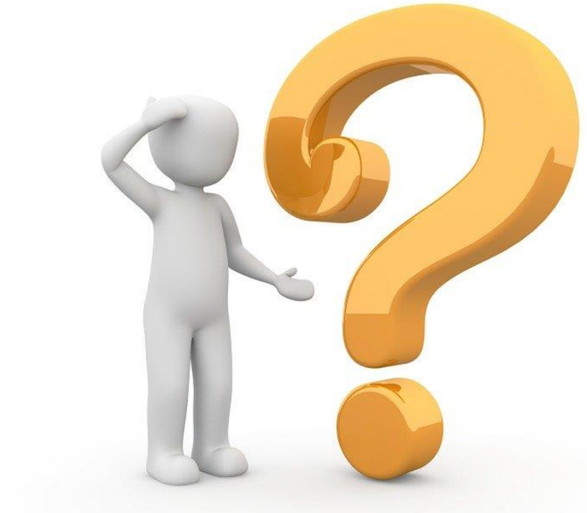

Footwear to prevent falls

- Closed with adjustable straps
- Adjusted size
- Soft material
- With non-slip soles
- Without heels

How and where to document a risk of falls

- Documentation is done by nurses in the EHR.
- Open dossier: Nursing assessement => To the right of «Habits / Needs»: click on «ST»
- Physicians also have access to the questionnaire and can make adjustments.

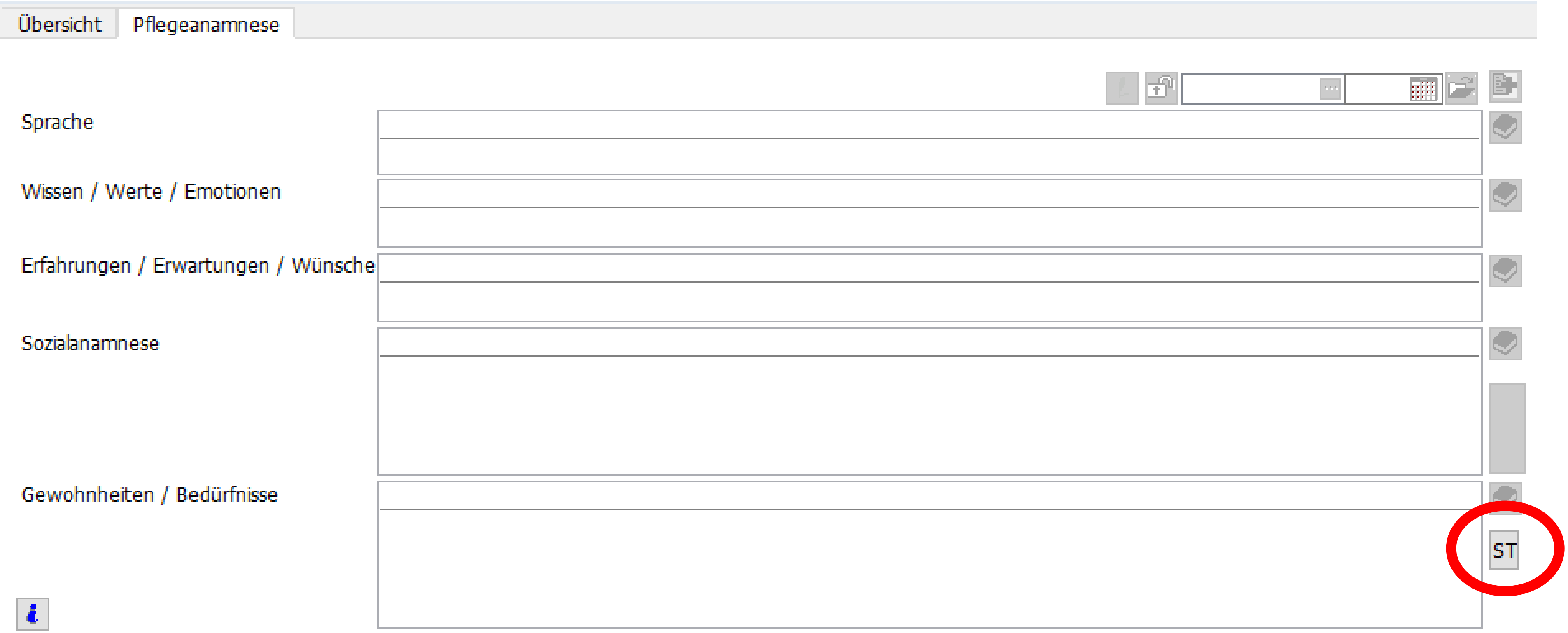

Documentation in the EHR

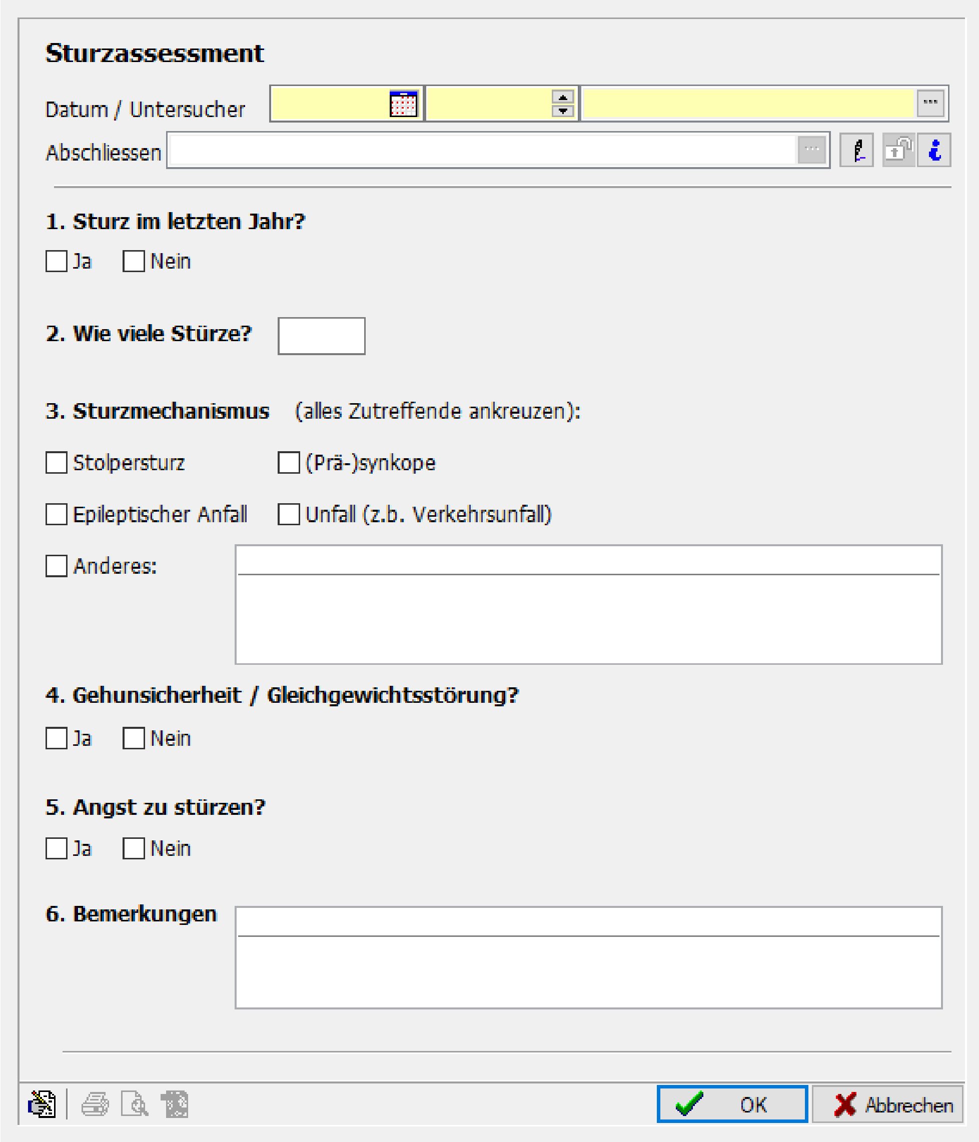

Sturzassessment

Datum / Untersucher

Abschliessen

1. Sturz im letzten Jahr?

☐ Ja ☐ Nein

2. Wie viele Stürze?

3. Sturzmechanismus (alles Zutreffende ankreuzen):

☐ Stolpersturz ☐ (Prä-)synkope

☐ Epileptischer Anfall ☐ Unfall (z.b. Verkehrsunfall)

☐ Anderes:

4. Gehunsicherheit / Gleichgewichtsstörung?

☐ Ja ☐ Nein

5. Angst zu stürzen?

☐ Ja ☐ Nein

6. Bemerkungen

OK Abbrechen

- Questions 1, 4 and 5 must always be answered
- Questions 2 and 3 only if “Yes” to question 1

Quiz 6

Question

When should the risk of falls be documented for the first time in the nursing dossier?

One answer is correct

1. After 5 days on the ward, when you know the patient better.
2. Upon entry
3. After physiotherapy assessment

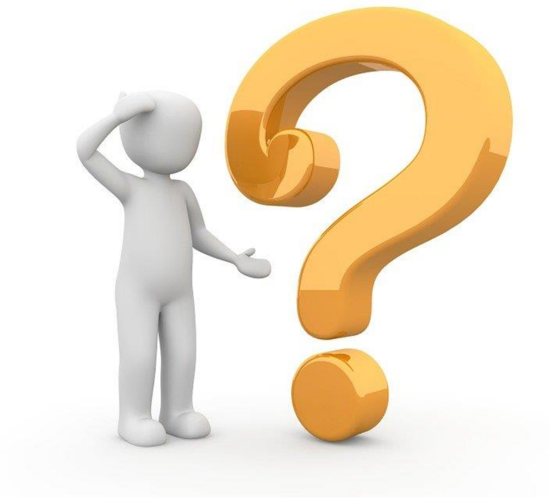

How and where to document a risk of falls

- Documentation is done by nurses in the EHR.
- Open dossier: Nursing assessement => To the right of «Habits / Needs»: click on «ST»
- Physicians also have access to the questionnaire and can make adjustments.

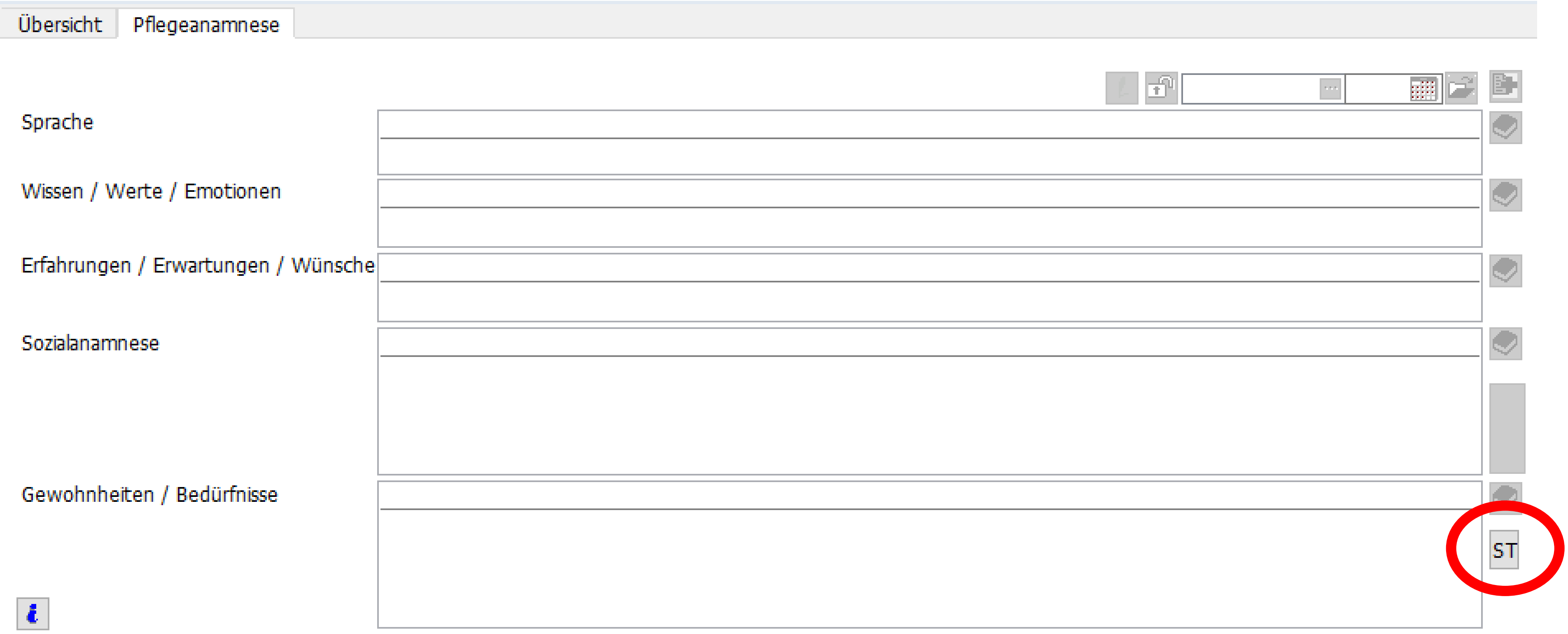

Documentation in the EHR

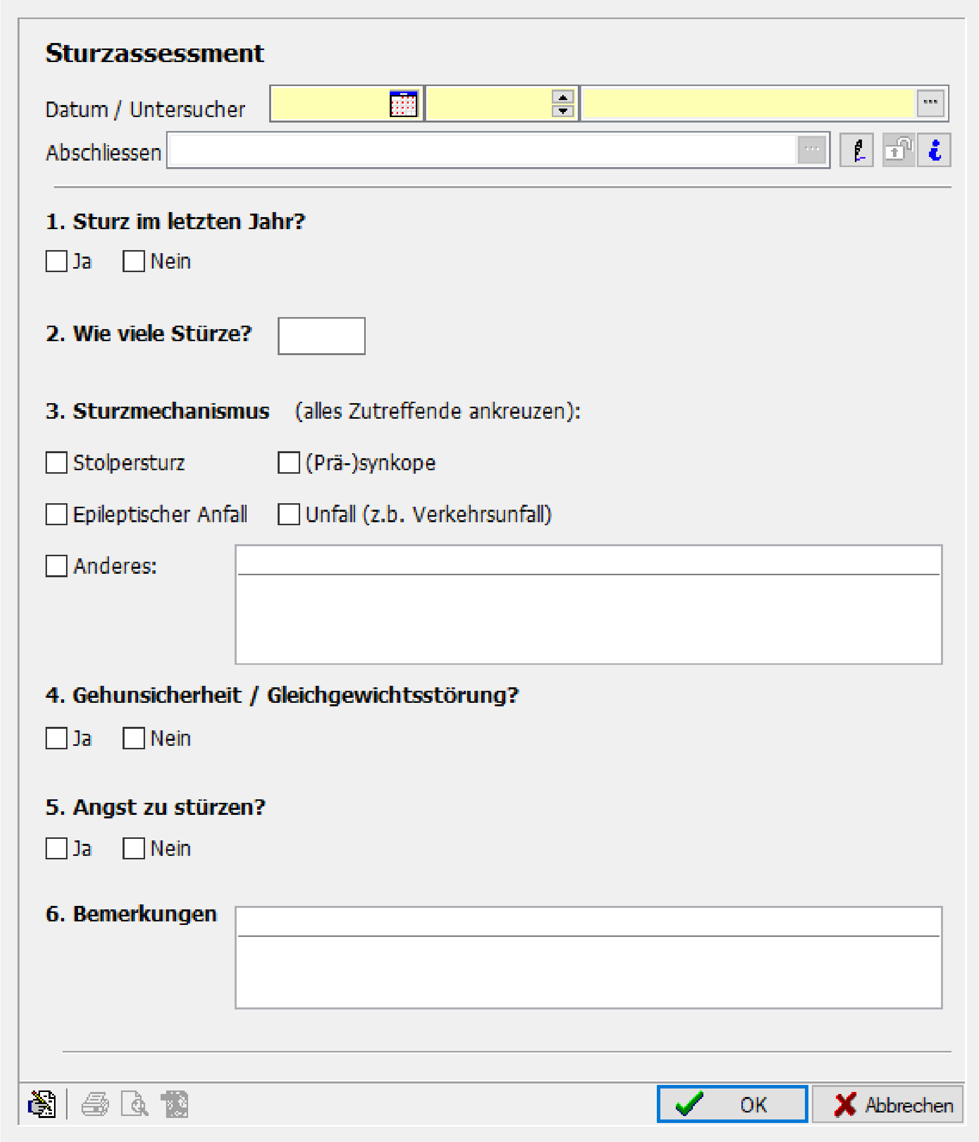

Sturzassessment

Datum / Untersucher

Abschliessen

1. Sturz im letzten Jahr?

☐ Ja ☐ Nein

2. Wie viele Stürze?

3. Sturzmechanismus (alles Zutreffende ankreuzen):

☐ Stolpersturz ☐ (Prä-)synkope

☐ Epileptischer Anfall ☐ Unfall (z.b. Verkehrsunfall)

☐ Anderes:

4. Gehunsicherheit / Gleichgewichtsstörung?

☐ Ja ☐ Nein

5. Angst zu stürzen?

☐ Ja ☐ Nein

6. Bemerkungen

OK Abbrechen

- Questions 1, 4 and 5 must always be answered
- Questions 2 and 3 only if “Yes” to question 1

Quiz 7

Question

Which medications increase the risk of falls?

Several answers are correct

A. Fentanyl
B. ACE-inhibitor
C. NSAID
D. Piperacilline/Tazobactam
E. Morphine

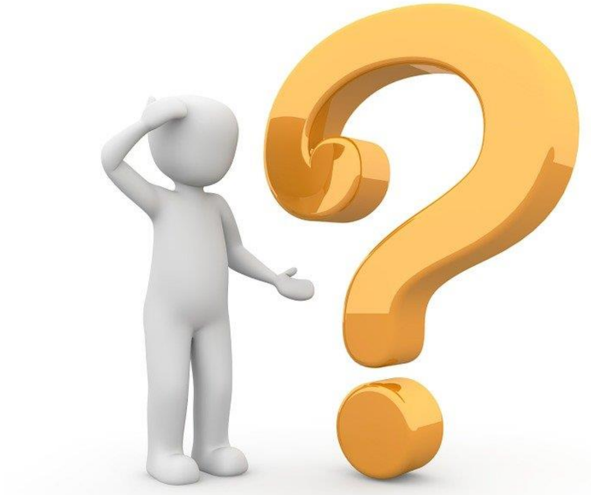

Medications that increase the risk of falls:

1. antipsychotics
2. antidepressants
3. anticholinergics (including drugs for irritable bladder)
4. antiepileptics
5. antihistamines
6. alpha blockers (antihypertensives / drugs for benign prostatic hyperplasia)
7. benzodiazepines & other sedatives
8. antihypertensives
9. vasodilators
10. diuretics
11. opioids

How and where to document a risk of falls

- Documentation is done by nurses in the EHR.
- Open dossier: Nursing assessement => To the right of «Habits / Needs»: click on «ST»
- Physicians also have access to the questionnaire and can make adjustments.

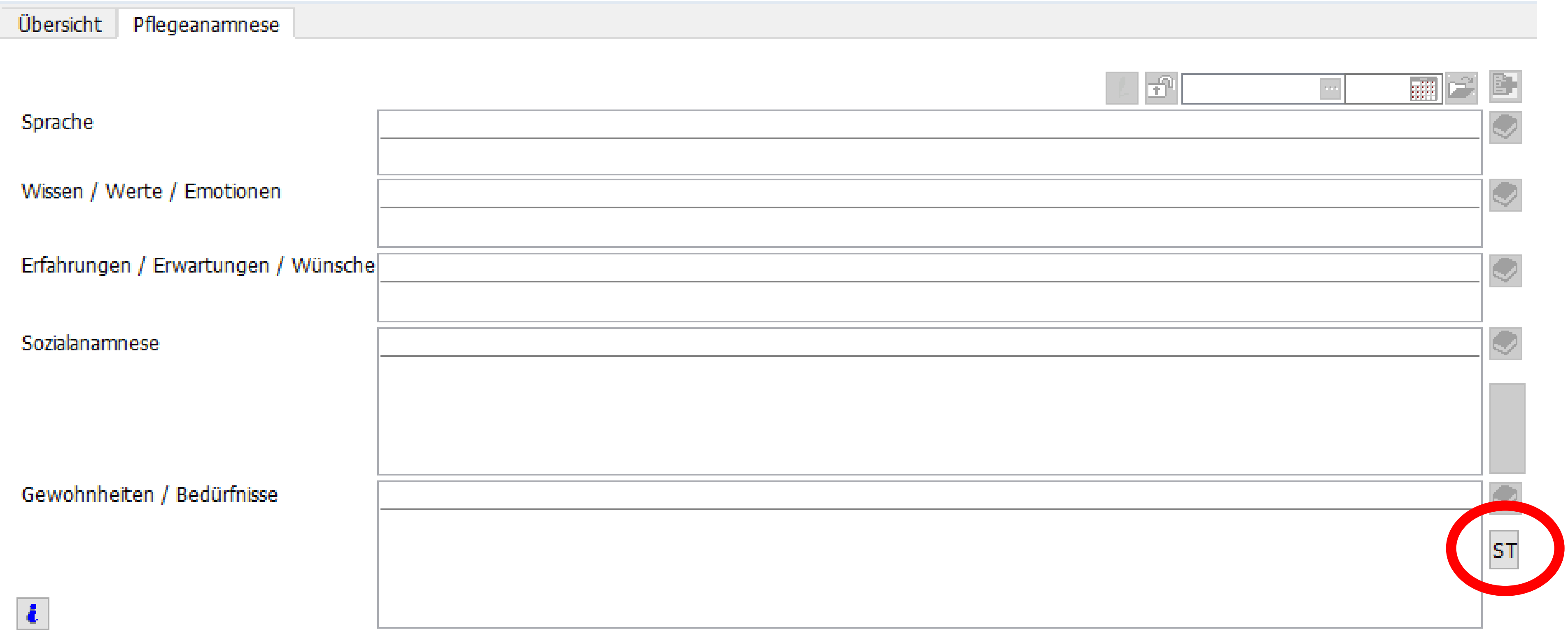

Documentation in the EHR

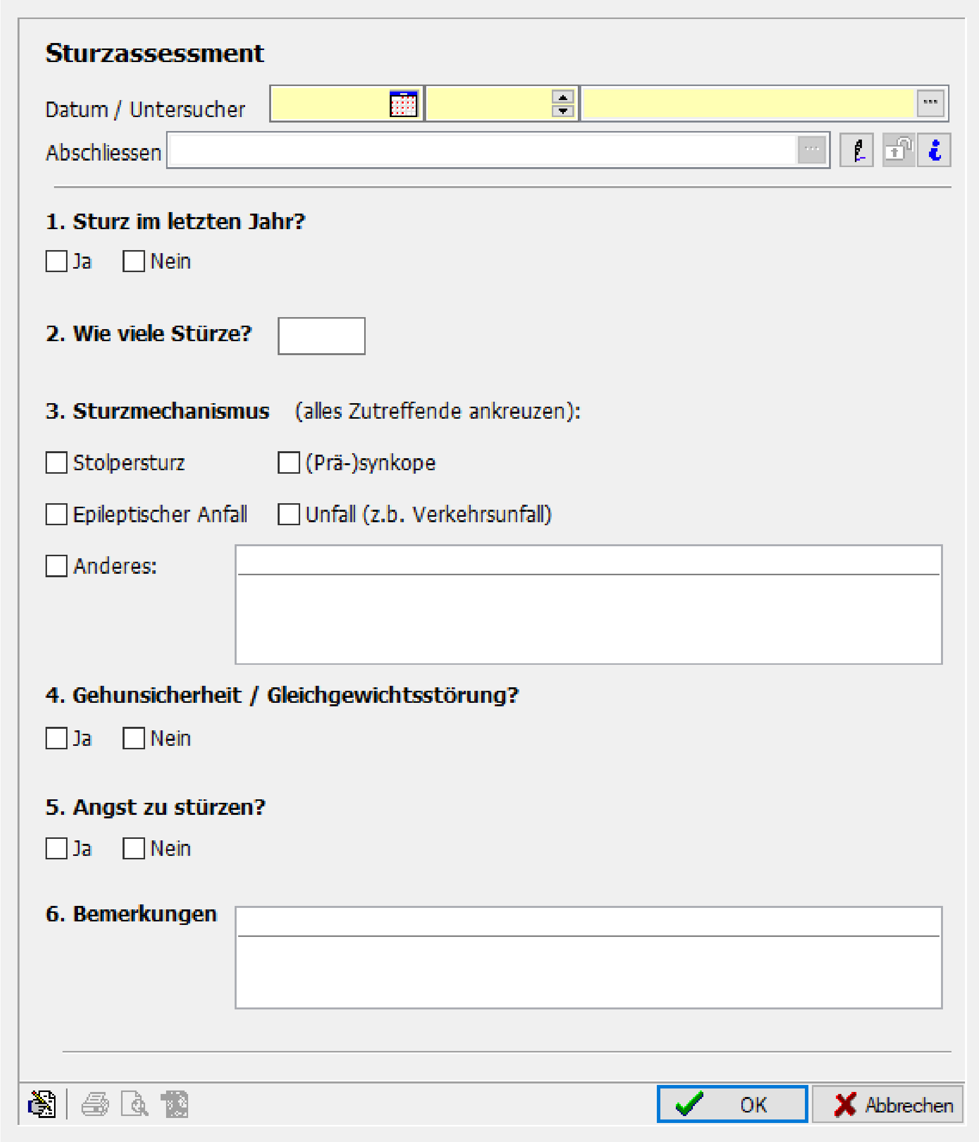

- Questions 1, 4 and 5 must always be answered
- Questions 2 and 3 only if “Yes” to question 1

Quiz 8

Question

What are risk factors for falls?

Several answers are correct

1. Parkinson's disease
2. A previous fall 8 months ago
3. Depression
4. Malnutrition

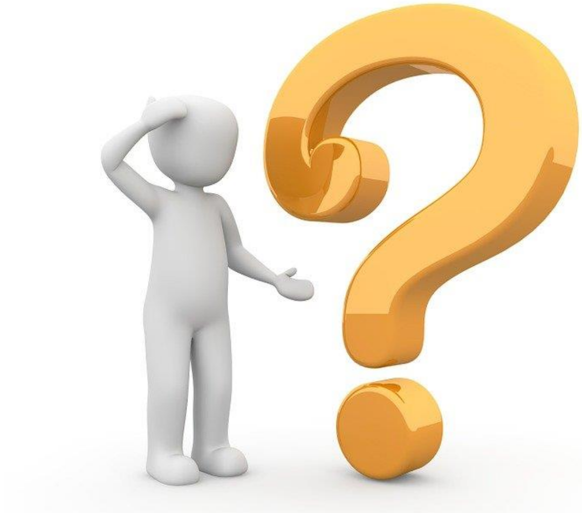

Predisposing risk factors

- Falls in the last 12 months
- Fear of falling
- Visual impairment
- Delirium, dementia, cognitive impairment
- Depression
- Epilepsy
- Hemiplegia
- Gait and balance disorders
- Parkinson's disease
- Joint pain
- Cardiac arrhythmias
- Orthostatic hypotension
- Syncope
- Malnutrition
- Urinary incontinence

Trigger risk factors

- Inappropriate use of walking aids
- Inappropriate shoes
- Environment (e.g. insufficient light) and change of environment
- Inadequate freedom-restricting measures
- Medications that increase the risk of falls

How and where to document a risk of falls

- Documentation is done by nurses in the EHR.
- Open dossier: Nursing assessement => To the right of «Habits / Needs»: click on «ST»
- Physicians also have access to the questionnaire and can make adjustments.

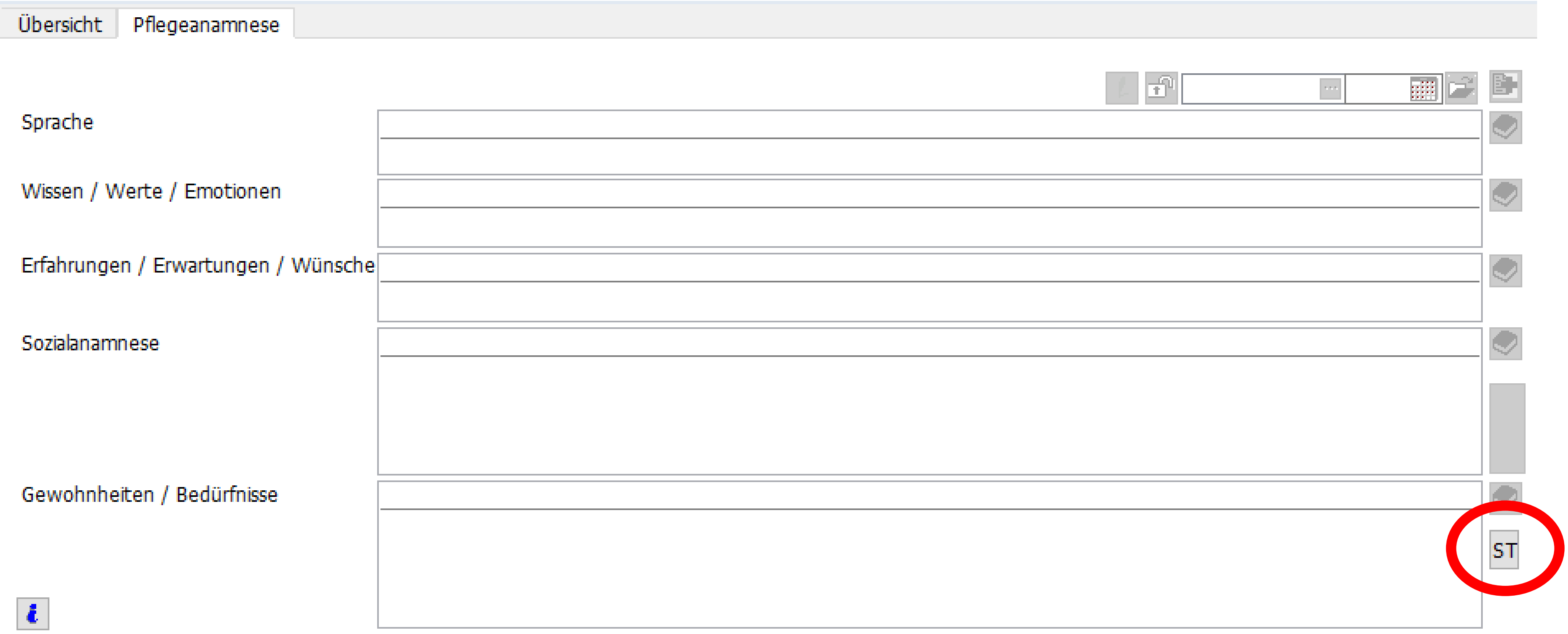

Documentation in the EHR

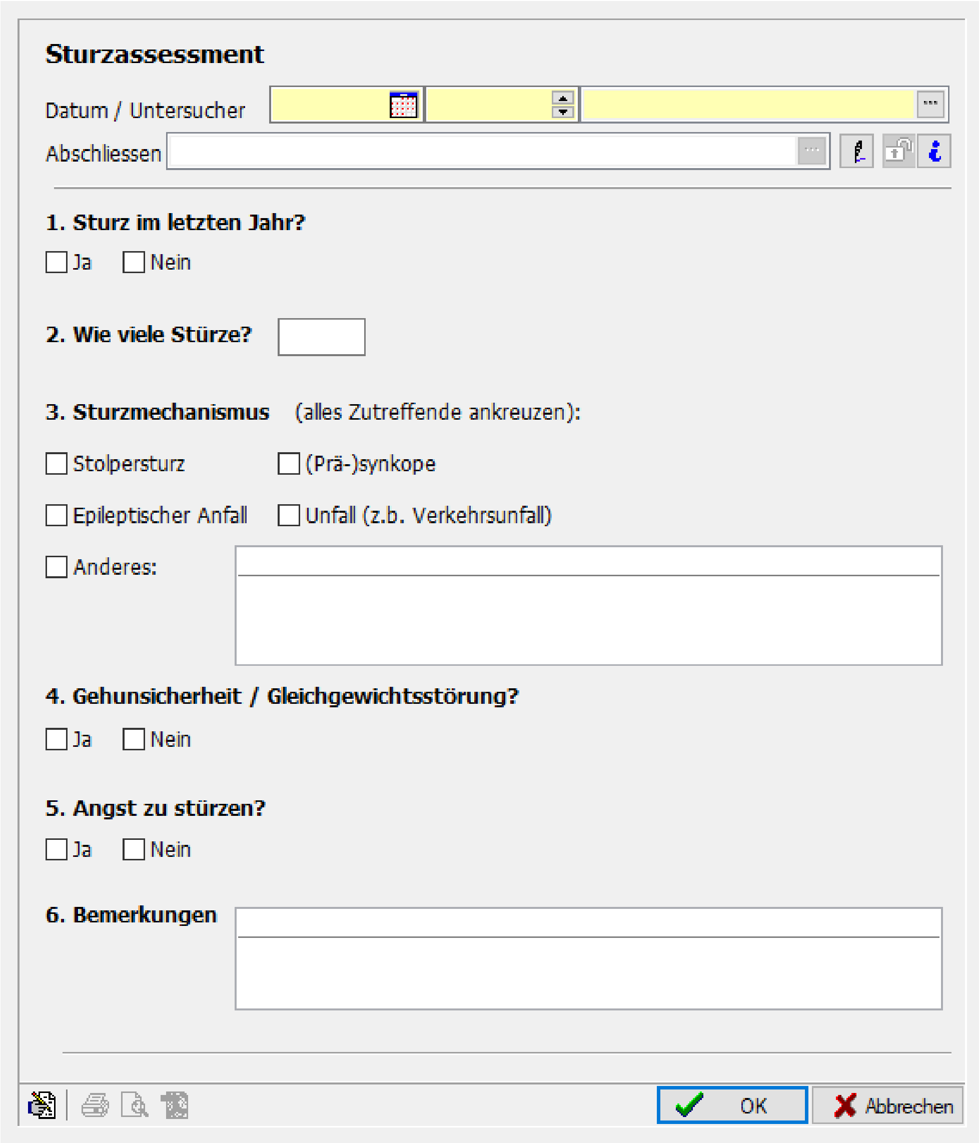

- Questions 1, 4 and 5 must always be answered
- Questions 2 and 3 only if “Yes” to question 1

Quiz 9

Question

What are measures for fall prevention?

Several answers are correct

A. Recommend closed shoes
B. Provide eyeglasses and an alarm system
C. Avoid leaving objects on the floor
D. Provide assistive devices

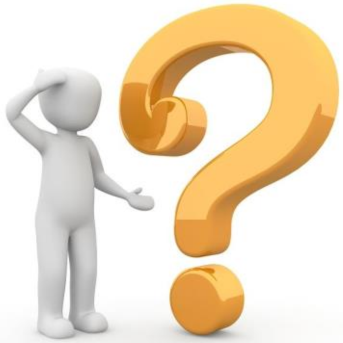

Measures to prevent falls

The measures to prevent falls must be tailored individually. Here are some measures:

- Encourage movement throughout the day without waiting for physiotherapy.
- Review of medication: active ingredient, indication, dosage, time of administration.
- Adapt food to nutritional needs.
- Evaluate hazards in the environment (light, objects on the ground).
- Provide an alarm system (e.g., patient bell).

Measures to prevent falls

- Podiatry and adapted shoes: Closed, with adjustable fasteners, correct size, soft material.
- Eyeglasses, telephone, assistive devices available.
- Toilet easy to reach.
- Inquire about living conditions so that the necessary adjustments can be organized before discharge home.
- Educate patients and their relatives: Ask about individual needs of patients and their relatives to prevent falls during and after hospitalization.

How and where to document a risk of falls

- Documentation is done by nurses in the EHR.
- Open dossier: Nursing assessement => To the right of «Habits / Needs»: click on «ST»
- Physicians also have access to the questionnaire and can make adjustments.

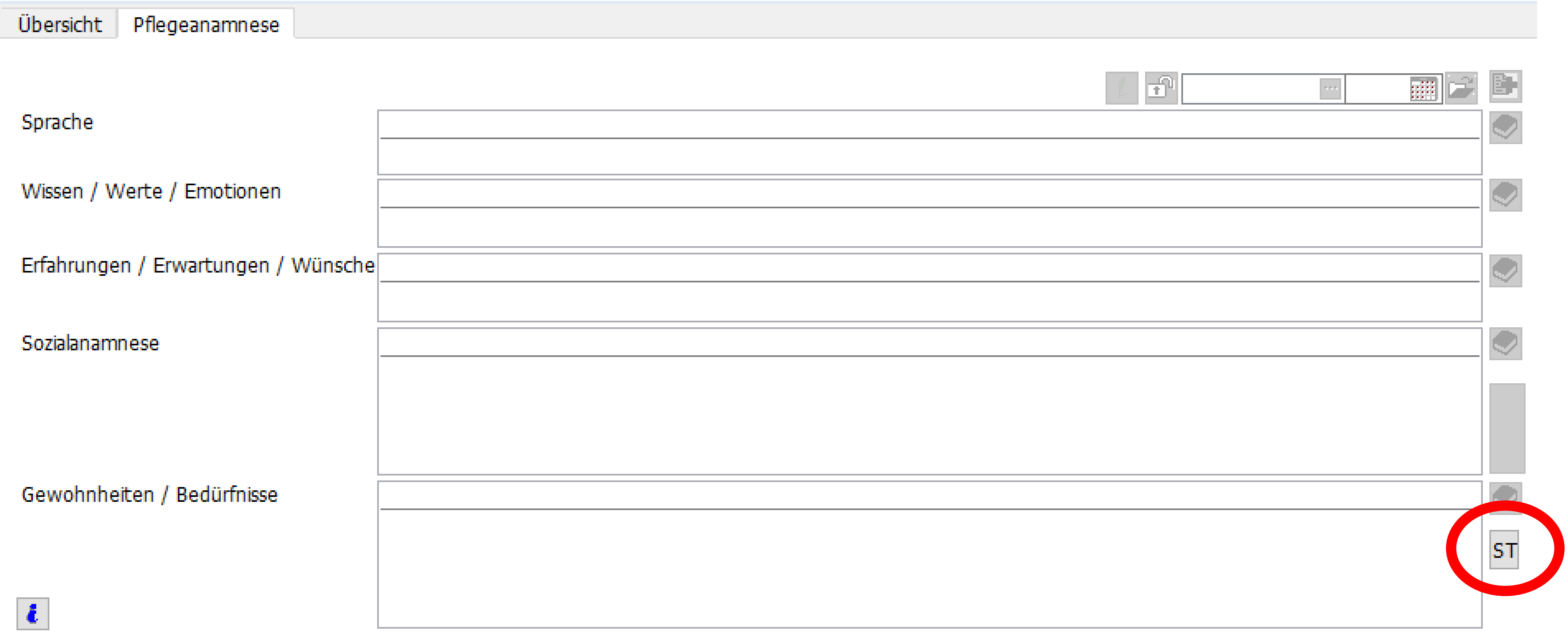

Documentation in the EHR

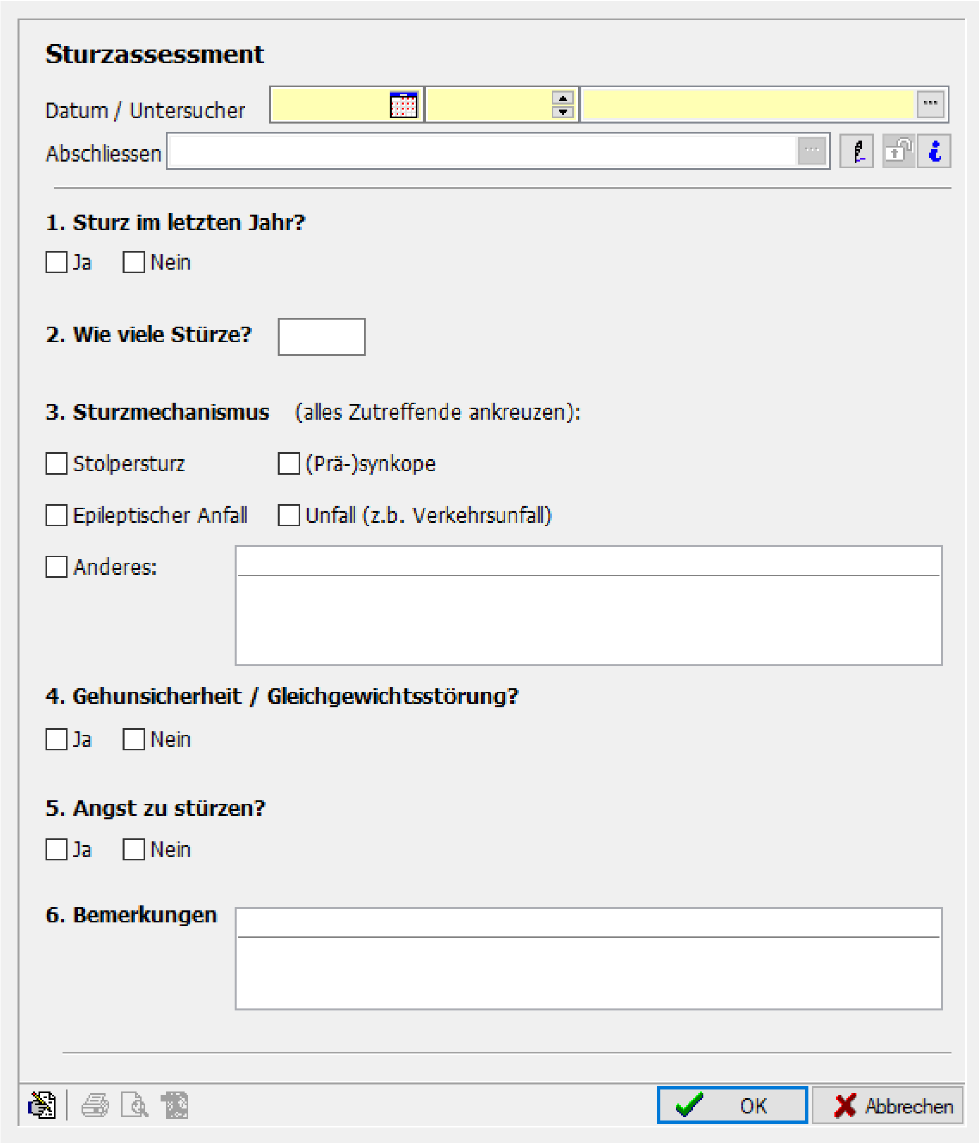

Sturzassessment

Datum / Untersucher

Abschliessen

1. Sturz im letzten Jahr?

☐ Ja ☐ Nein

2. Wie viele Stürze?

3. Sturzmechanismus (alles Zutreffende ankreuzen):

☐ Stolpersturz ☐ (Prä-)synkope

☐ Epileptischer Anfall ☐ Unfall (z.b. Verkehrsunfall)

☐ Anderes:

4. Gehunsicherheit / Gleichgewichtsstörung?

☐ Ja ☐ Nein

5. Angst zu stürzen?

☐ Ja ☐ Nein

6. Bemerkungen

OK Abbrechen

- Questions 1, 4 and 5 must always be answered
- Questions 2 and 3 only if “Yes” to question 1
