# Supplementary material for: A pre-post intervention study to improve fall risk assessment in older hospitalised adults: the STROLL study
Source: BMC Geriatr. 2025 Nov 29;26:171. doi: 10.1186/s12877-025-06817-5 (PMC12882572; doi:10.1186/s12877-025-06817-5)
Supplement: Supplementary file 4 — Additional file 4. Reminder quizzes for nurses (pdf format). [file 12877_2025_6817_MOESM4_ESM.pdf]

The most important thing is to stand up again!

Fall prevention through three key points

Learning Goals

After completion of this e-learning, you are able to:

- Recognize and document falls that happened during the past 12 months.
- Identify the risk of future falls.
- Suggest preventive measures to hospitalized patients and their relatives.

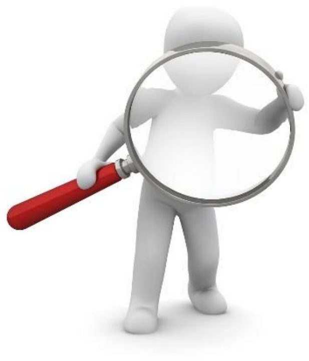

Content

- Fall risk factors in older persons
- Fall risk assessement
- Fall prevention

Recognize, document and prevent falls

Who is responible? ALL of us!

- Patients and their relatives
- Nurses
- Nursing assistants
- Physicians
- Physiotherapists

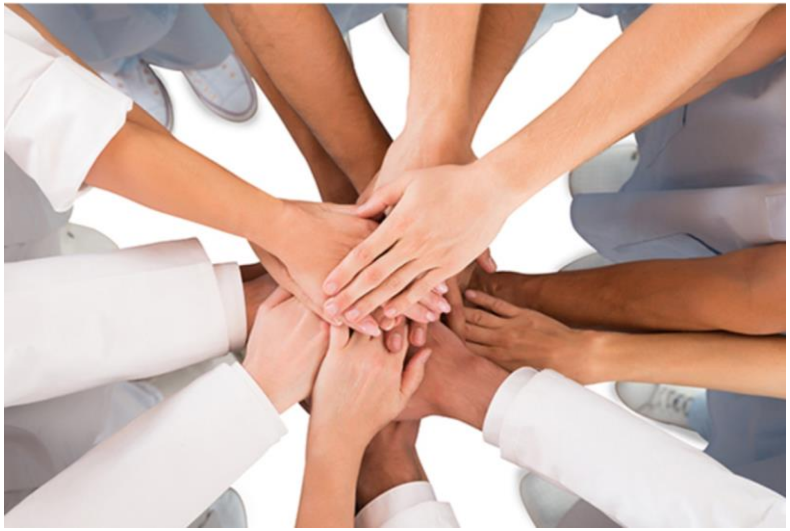

Falls, a short summary

Consequence of gait disorders

Event after which the person suddenly finds themselves on the ground

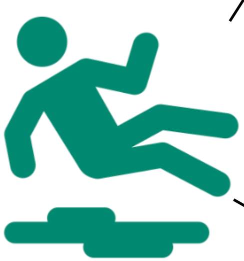

Often multifactorial and preventable

Risk increases with age

Influence on quality of life and physical health

Annual costs of two billion Swiss francs

Influence of age on the risk of falls

- The risk of falls increases exponentially after the age of 65:
  - **35%** of adults aged **≥80 years** have fallen in the past 12 months

Question 1

What are risk factors for falls?

Several answers are correct.

1. Treatment with tamsulosin.
2. A fall at home two weeks ago.
3. Treatment with lorazepam.
4. Fear of falling.
5. Furosemide intravenous.

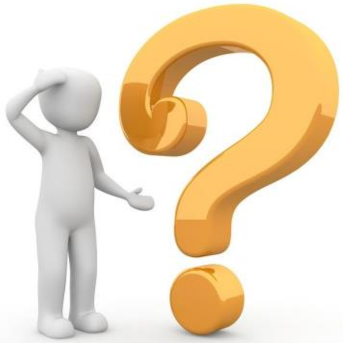

Answer to question 1

What are risk factors for falls?

All answers are correct.

1. correct!
2. correct!
3. correct!
4. correct!
5. correct!

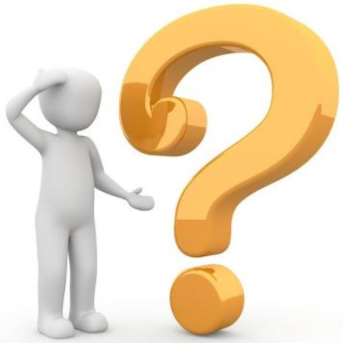

Risk factors and recommended measures

- Falls in the past 12 months: Assess and document systematically.
- Fear of falling: Promote mobilization, prescribe physiotherapy and add the comment «fear of falling».
- Inadequate freedom-restricting measures: Evaluate the need for these measures during medical rounds.
- Inappropriate assistive devices: Evaluate with physiotherapy.
- Medications that increase risk of falls: Avoid such prescriptions during hospitalization and review pre-existing medications.

Medications that increase the risk of falls:

1. antipsychotics
2. antidepressants
3. anticholinergics (including medication against irritable bladder)
4. antiepileptics
5. antihistamines
6. alpha blockers (as antihypertensive or against prostate hyperplasia)
7. hypnotics & sedatives
8. antihypertensives
9. vasodilators
10. diuretics
11. opioids

Question 2

The following questions are important to ask. Which of these questions should be **prioritized** upon admission in order to avoid a fall during and after hospitalization?

Only one answer is correct

1. Do you need to climb stairs to reach your apartment?

2. Did you fall in the past 12 months? If so, how often?

3. Do you need a walking aid?

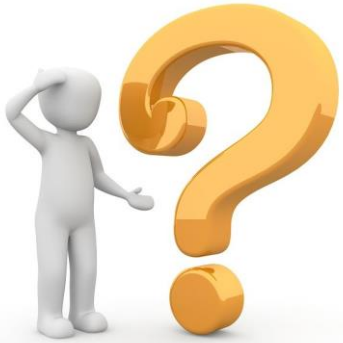

Answer to question 2

The following questions are important to ask. Which of these questions should be **prioritized** upon admission in order to avoid a fall during and after hospitalization?

1. This answer is correct but it’s not the first question which should be asked.

2. Correct!

3. This answer is correct but it’s not the first question which should be asked.

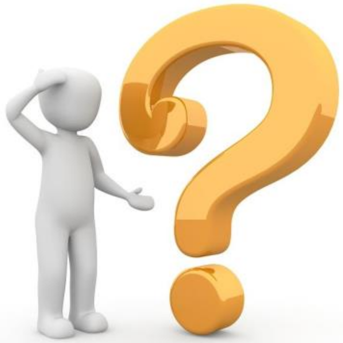

How can the risk of falls be recognized?
3 Key aspects

- Systematic questioning of individuals aged ≥65 years upon admission
- Did you **fall** in the past 12 months?

  if yes

- Details about the fall: How often and how did it happen?

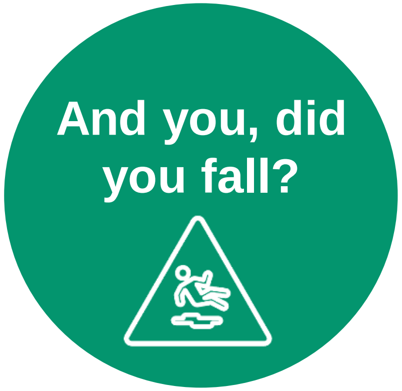

Documentation – discharge report

The discharge report must include the following:

- Diagnosis of “gait and balance disorders” with specification “with/without a fall”.

Including:

- Fall risk factors
- Home ressources and suggestions for improvement of home environment
- Adjustments of medication

Documentation carried out by nurses

- Picture and text to explain documentation in electronic health record

How and where to document?

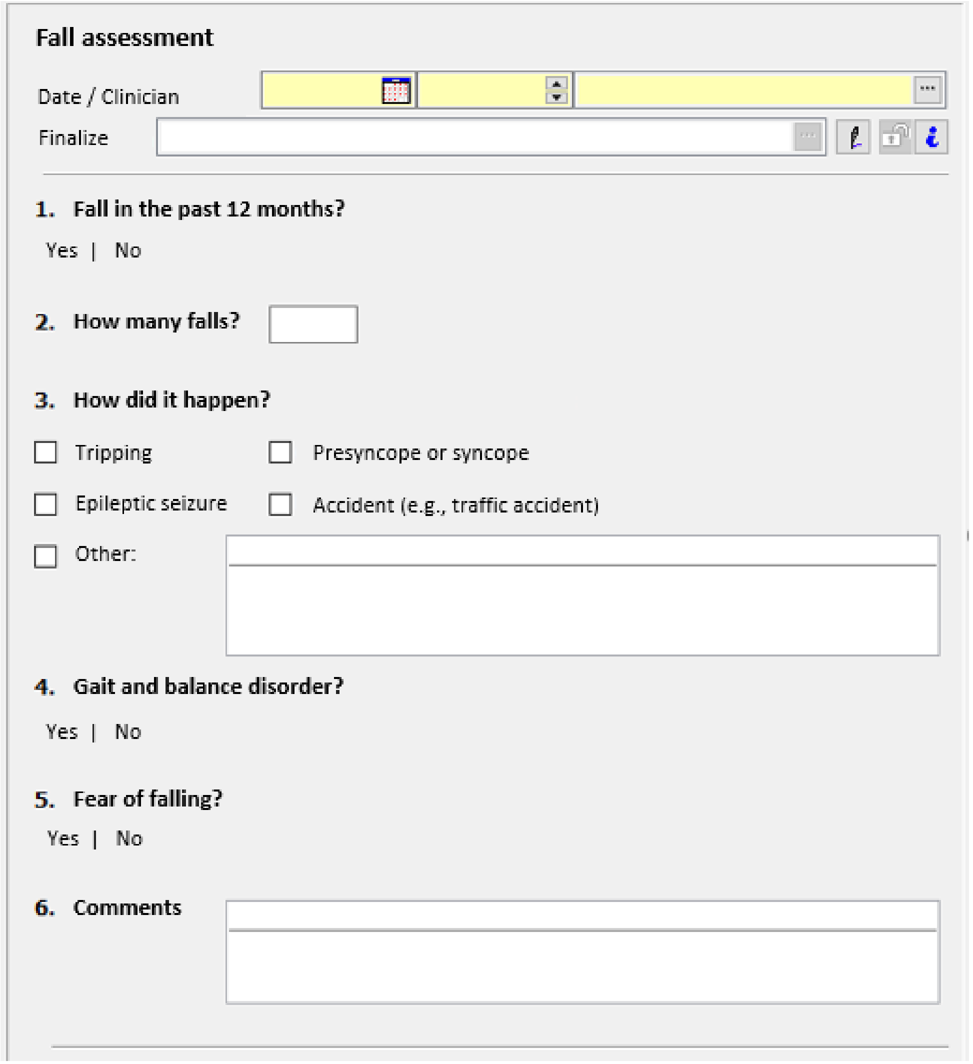

Fall assessment

Date / Clinician

Finalize

1. Fall in the past 12 months?

Yes | No

2. How many falls?

3. How did it happen?

- [ ] Tripping
- [ ] Presyncope or syncope
- [ ] Epileptic seizure
- [ ] Accident (e.g., traffic accident)
- [ ] Other:

4. Gait and balance disorder?

Yes | No

5. Fear of falling?

Yes | No

6. Comments

- Questions 1, 4 and 5 must always be answered
- Questions 2 and 3 only if “yes” to question 1

Question 3

Which of these measures can prevent a fall of older hospitalized persons?

Several answers are correct.

1. A urinary catheter that reduces movement to the bathroom.
2. Side rails to prevent a patient from standing up.
3. Not changing pre-existing medication.
4. Encouraging exercise during the rounds, independently of physiotherapy.
5. Not prescribing benzodiazepines.

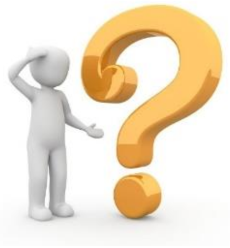

Answer to question 3

Which of these measures can prevent a fall of older hospitalized persons?

Several answers are correct.

1. This is not a measure of fall prevention.
2. This is not a measure of fall prevention.
3. This is not a measure of fall prevention, the current medication should always be reviewed.
4. Correct!
5. Correct!

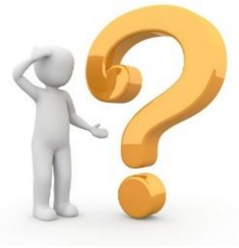

Preventive measures: Checking the medication

- **Indication:** Checking the necessity of therapy (e.g., according to STOPP/START criteria) during hospitalization
- **Dosage**
- **Timing of intake**
- **Route of administration**
- **Avoiding therapeutic cascade** = prescribing new medications for side effects of other medications.

An important brochure

Independent until old age

Live, move, stay mobile

Swiss Council for Accident Prevention

bfu.ch

- Available online and on the ward
- Contents:
  - Tricks
  - Self-assessment of fall risk
  - Checklist

The most important tricks

- Are you worried that you might fall? Then contact your primary care provider.
- By furnishing your home in an age-appropriate manner, you make everyday life easier and prevent falls.
- Daily exercise and a healthy diet contribute to preserving mobility and independence.

Don’t forget!

Early prevention thanks to one question that should be documented systematically:

Did you fall in the last 12 months?

It affects all of us! YOU are the key!

And you, did you fall?
